# Supplementary material for: Post-polymerisation functionalisation of conjugated polymer backbones and its application in multi-functional emissive nanoparticles
Source: Nat Commun. 2018 Aug 13;9:3237. doi: 10.1038/s41467-018-05381-4 (PMC6089984; doi:10.1038/s41467-018-05381-4)
Supplement: Supplementary file 1 — Supplementary Information [file 41467_2018_5381_MOESM1_ESM.pdf]

Quantitative post-polymerisation functionalisation of conjugated polymer backbones and its application in multi-functionalised semiconducting polymer nanoparticles

Creamer et al.

Table of Contents

Supplementary Figures 3
Supplementary Discussion 43
Calculation of FRET efficiency from PL and TCSPS data on SPN-N_3 43
Supplementary Methods 48
Precursor monomers of polymers P1-P6 48
Synthesis of polymers P1-P5 49
Synthesis of P1-SR, P2-Silane, P2-Copolymer, P3-SR, P4-SR, P5-SR and P6-SR 52
Substitution of P2-F with increasing thiol content 55
Synthesis of P2-N_3, P2-Alkene, P2-OR and P2-PEG 56
Supplementary Tables 58
Supplementary References 59

Supplementary Figures

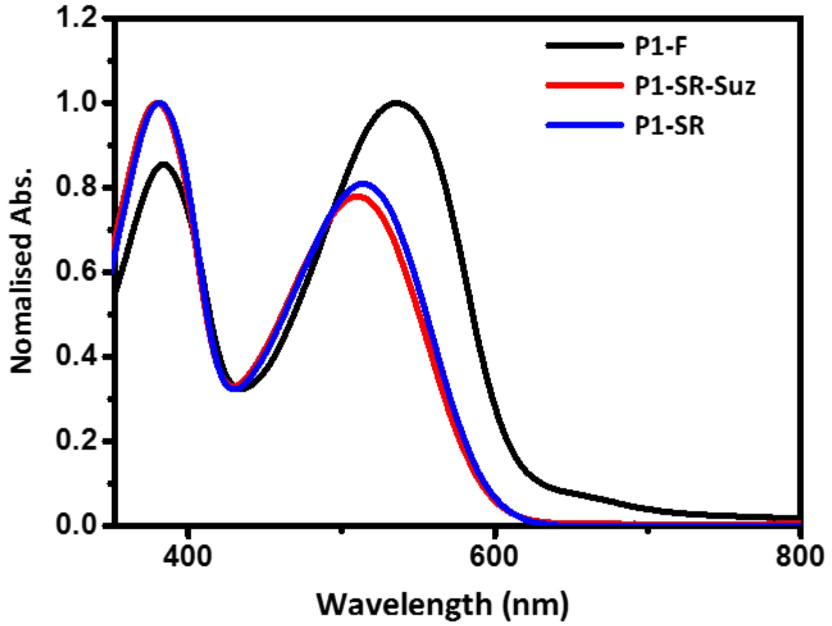

Supplementary Figure 1: Normalised UV-Vis spectra of P1 derivatives. Normalised UV-Vis spectra of **P1-F** (black), **P1-SR-Suz** (red) and **P1-SR** (blue) in Chlorobenzene solution.

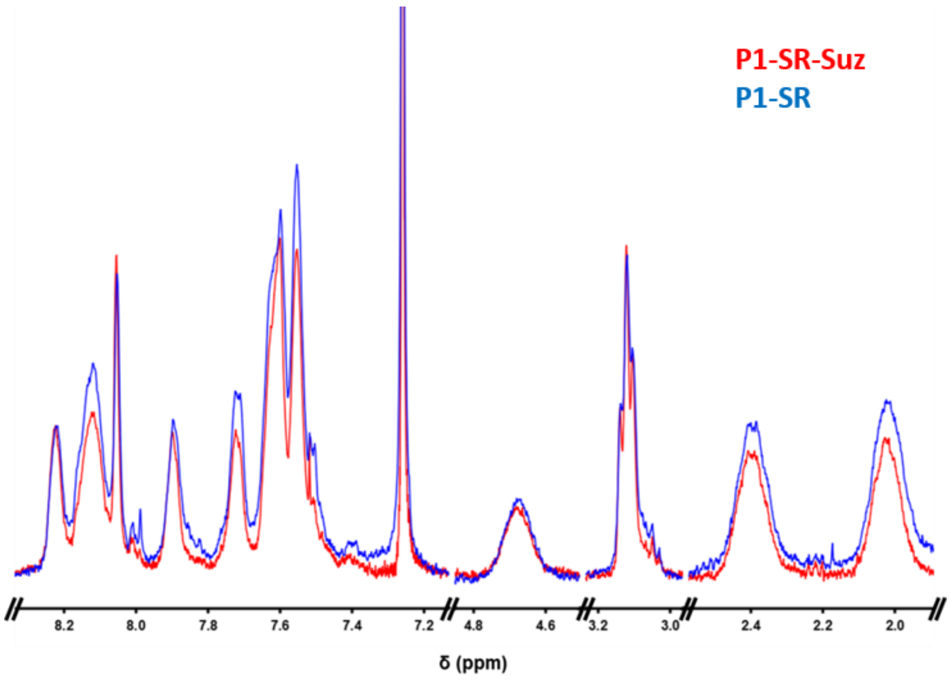

Supplementary Figure 2: NMR spectra of P1-SR-Suz and P1-SR. Overlayed ^{1}H NMR of **P1-SR-Suz** (red line) and **P1-SR** (blue line), trimmed to show important chemical shift regions, in $CDCl_3$ at 400 MHz.

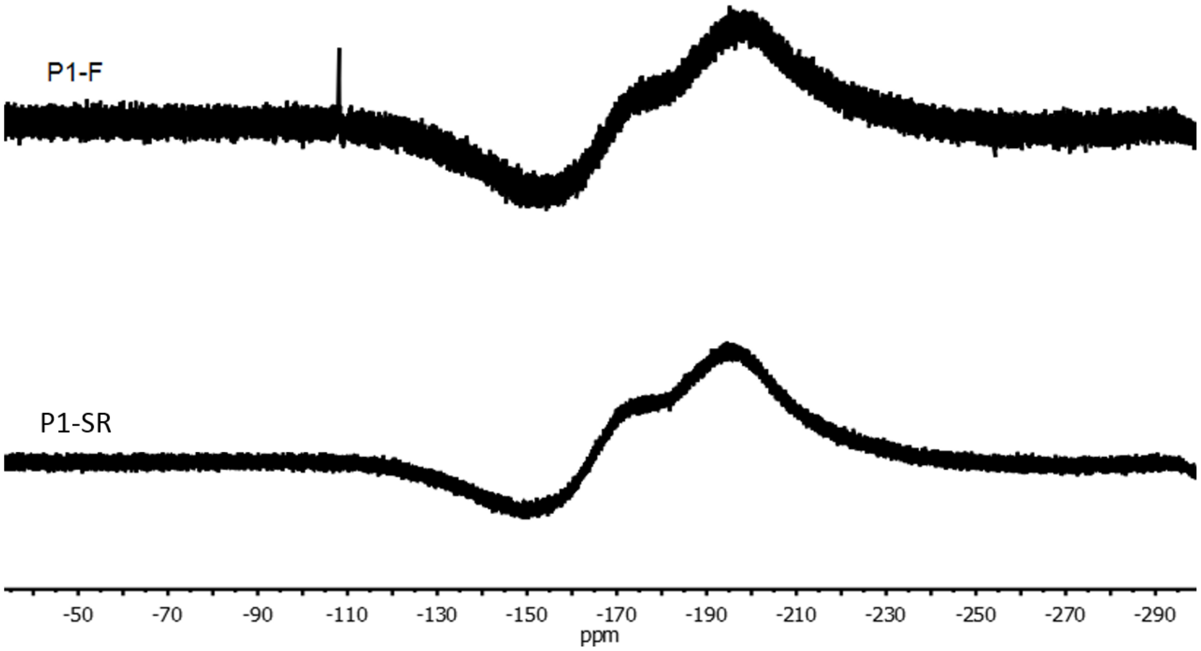

Supplementary Figure 3: ^{19}F NMR of P1-F and P1-SR. Stacked ^{19}F NMR of **P1-F** and **P1-SR** in $CDCl_3$ at 400 MHz.

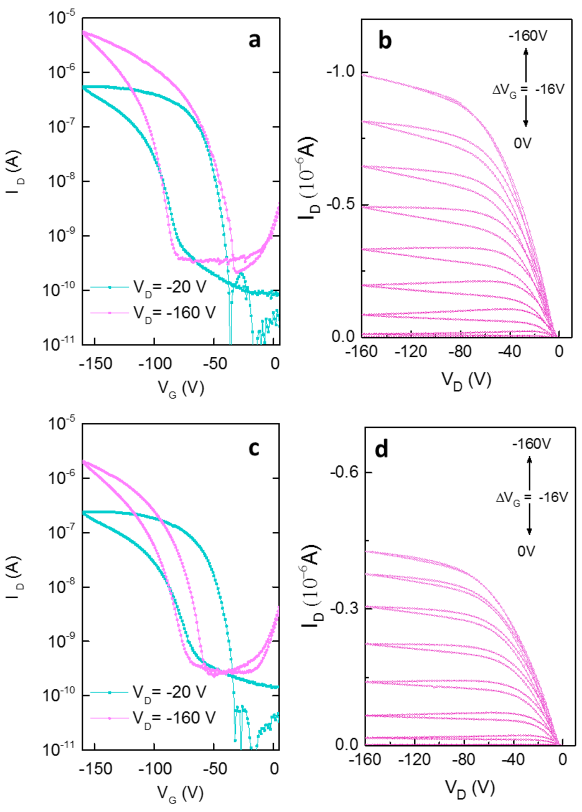

Supplementary Figure 4: Transistor characteristics for P1-SR-Suz and P1-SR. Representative transfer (**a**,**c**) and output (**b**,**d**) characteristics of **P1-SR-Suz** (**a**,**b**) and **P1-SR** (**c**,**d**) in bottom contact, bottom gate transistors with channel length 10 µm and with 1500 µm.

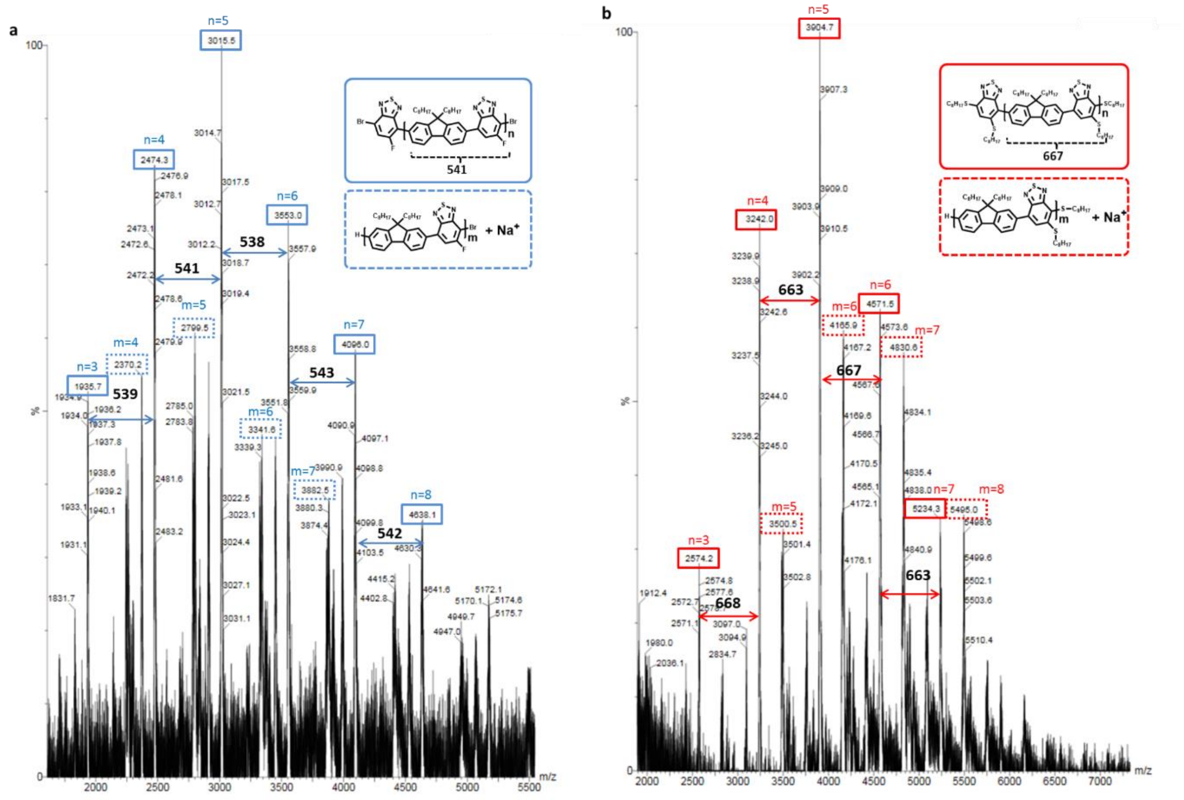

Supplementary Figure 5: MALDI-TOF spectra of P2-F and P2-SR. Matrix assisted laser deposition/ionisation time of flight (MALDI-TOF) mass spectra of low M_W fractions of a) **P2-F** and b) **P2-SR** (**P1-F** reacted with 1-octanethiol).

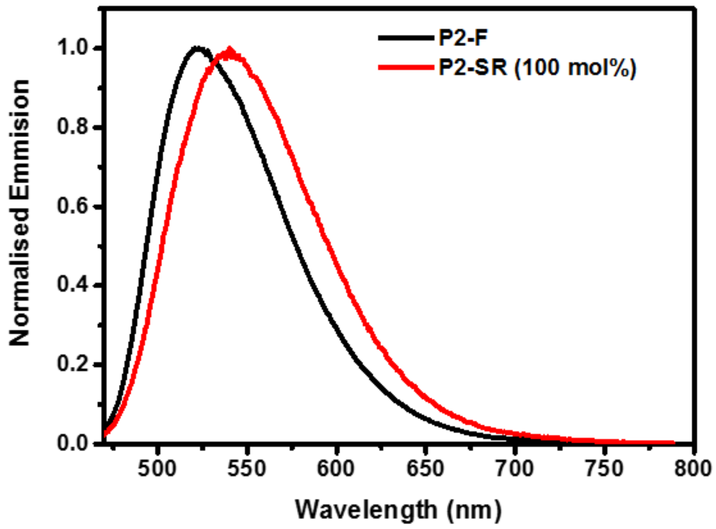

Supplementary Figure 6: PL emission spectra of P2-F and P2-SR. Normalised emission spectra of **P2-F** and fully substituted **P2-SR** in THF. Samples pulsed at 450 nm.

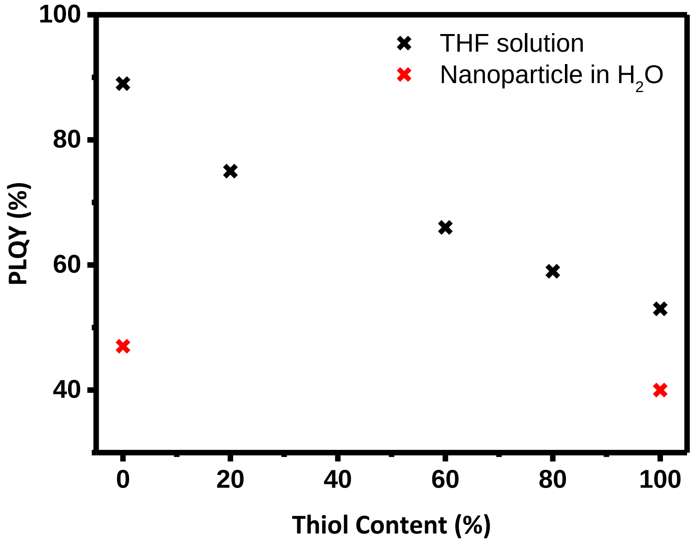

Supplementary Figure 7: PLQY of P2-F with increasing percentage of thiol content. Photoluminescence quantum yield (PLQY) of **P2-F** samples dissolved in THF (black crosses) and nanoparticle suspensions in water (red crosses), with increasing percentage of thiol content.

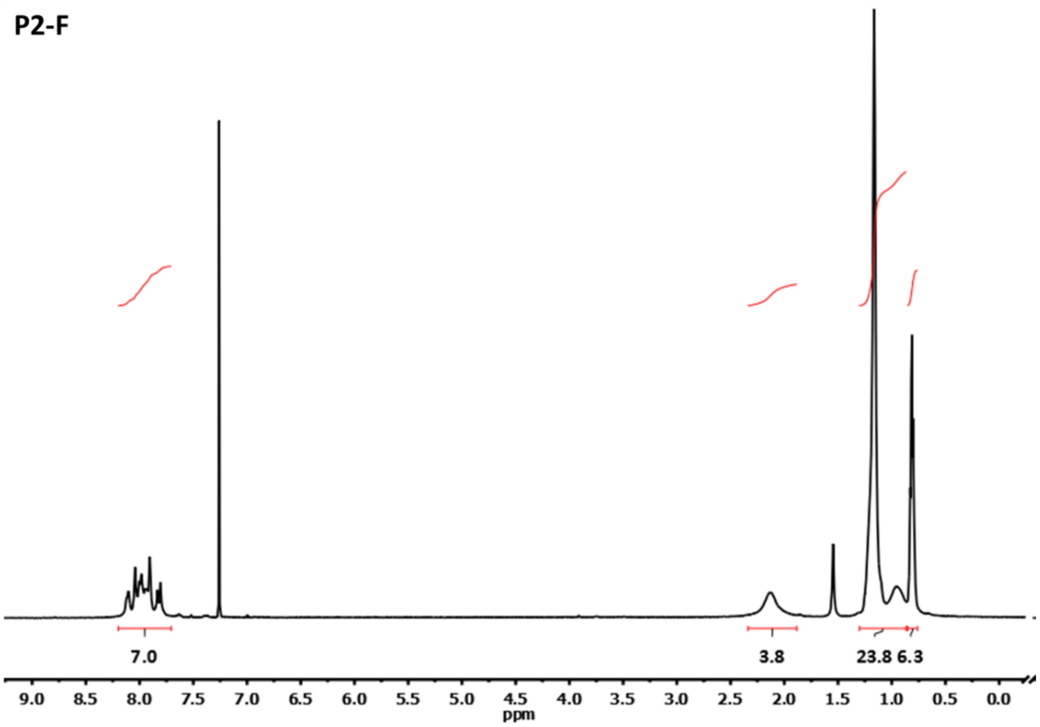

Supplementary Figure 8: ^{1}H NMR of P2-F. ^{1}H NMR of polymer **P2-F** in $CDCl_3$ at 400 MHz.

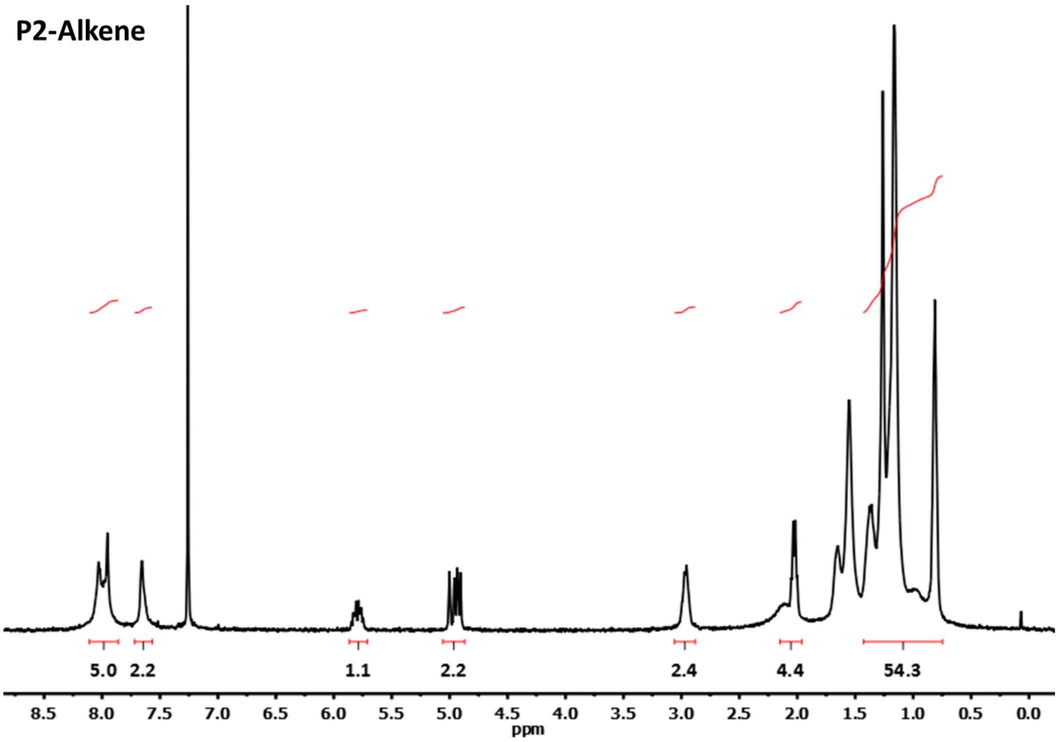

Supplementary Figure 9: ^{1}H NMR of P2-Alkene. ^{1}H NMR of polymer **P2-Alkene** in $CDCl_3$ at 400 MHz.

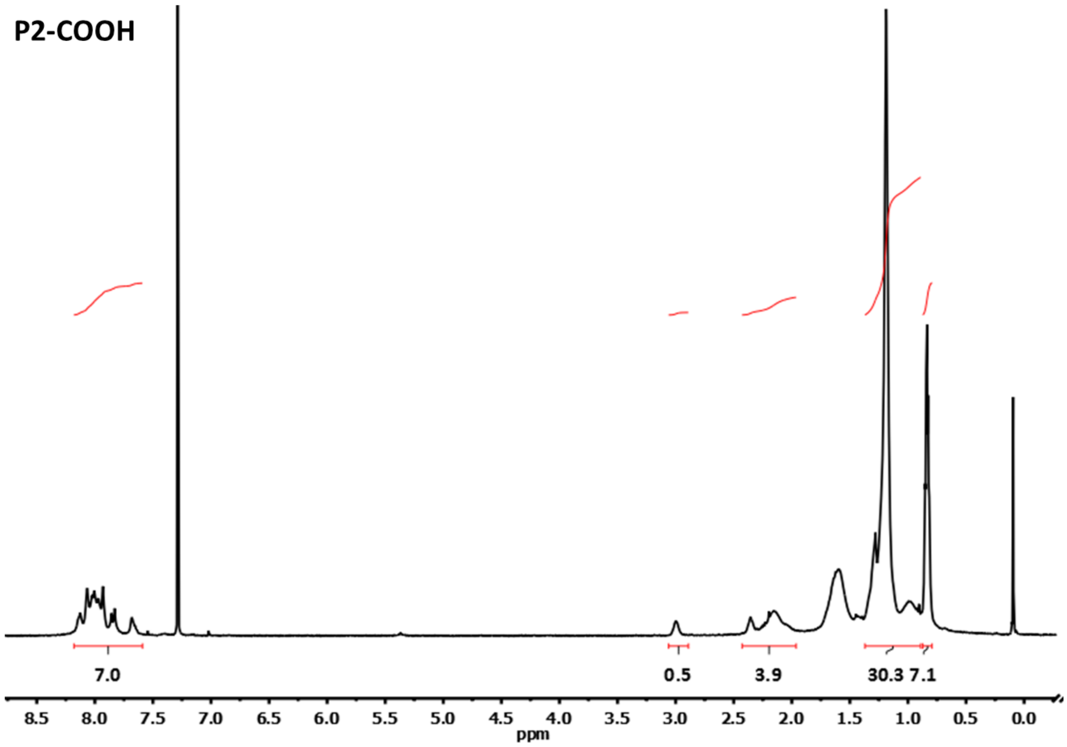

Supplementary Figure 10: ^{1}H NMR of P2-COOH. ^{1}H NMR of polymer **P2-COOH** in $CDCl_3$ at 400 MHz.

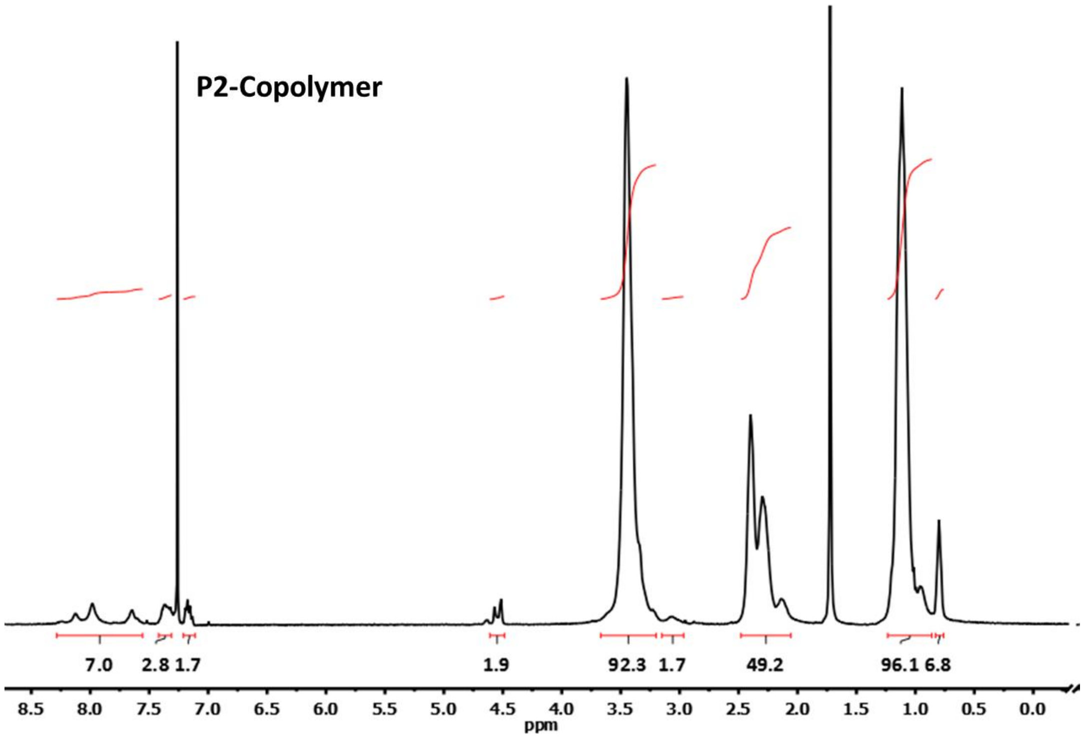

Supplementary Figure 11: ^{1}H NMR of P2-Copolymer. ^{1}H NMR of polymer **P2-Copolymer** in $CDCl_3$ at 400 MHz.

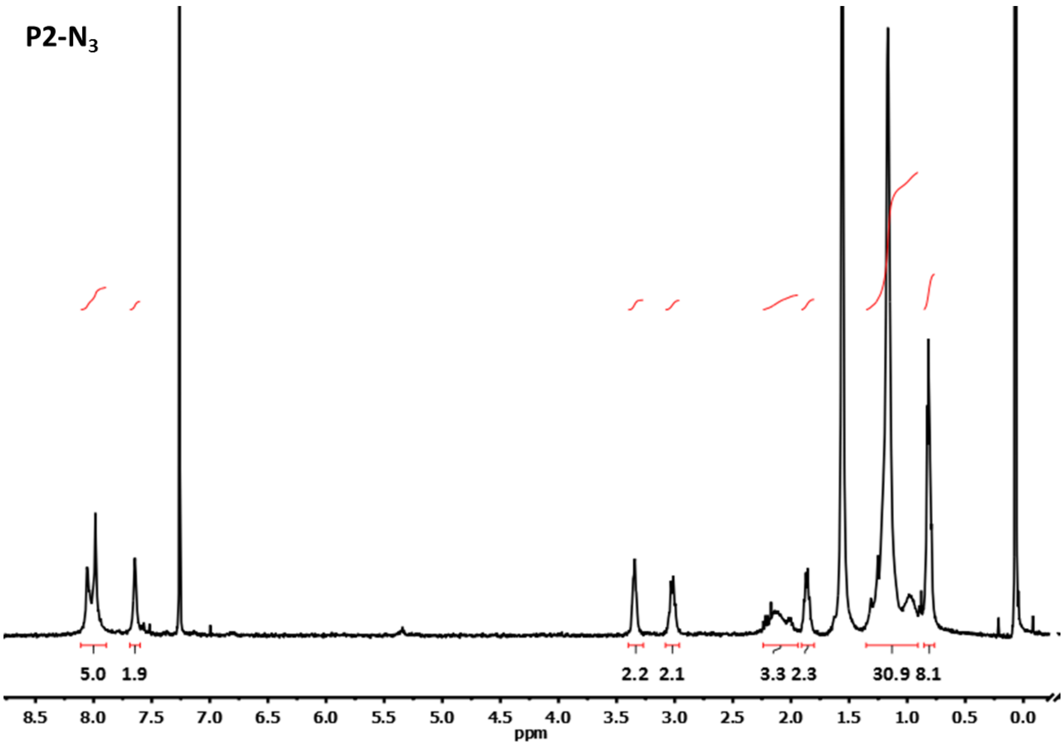

Supplementary Figure 12: ^{1}H NMR of P2-N$_3$. ^{1}H NMR of polymers **P2-N$_3$** in $CDCl_3$ at 400 MHz.

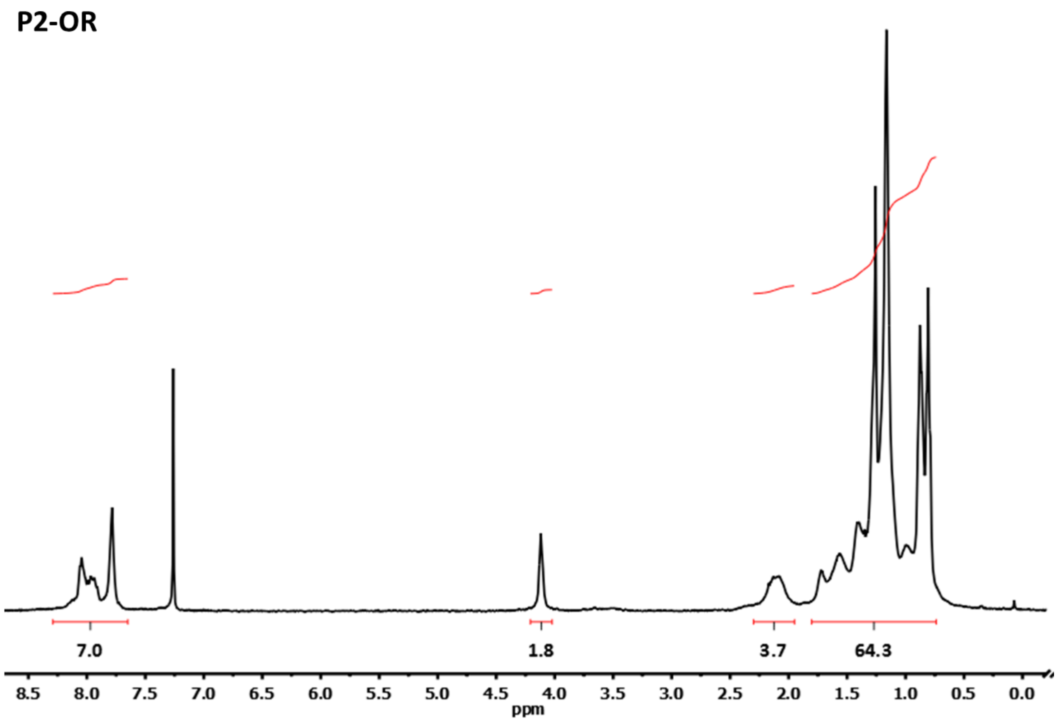

Supplementary Figure 13: ^{1}H NMR of P2-OR. ^{1}H NMR of polymer **P2-OR** in $CDCl_3$ at 400 MHz.

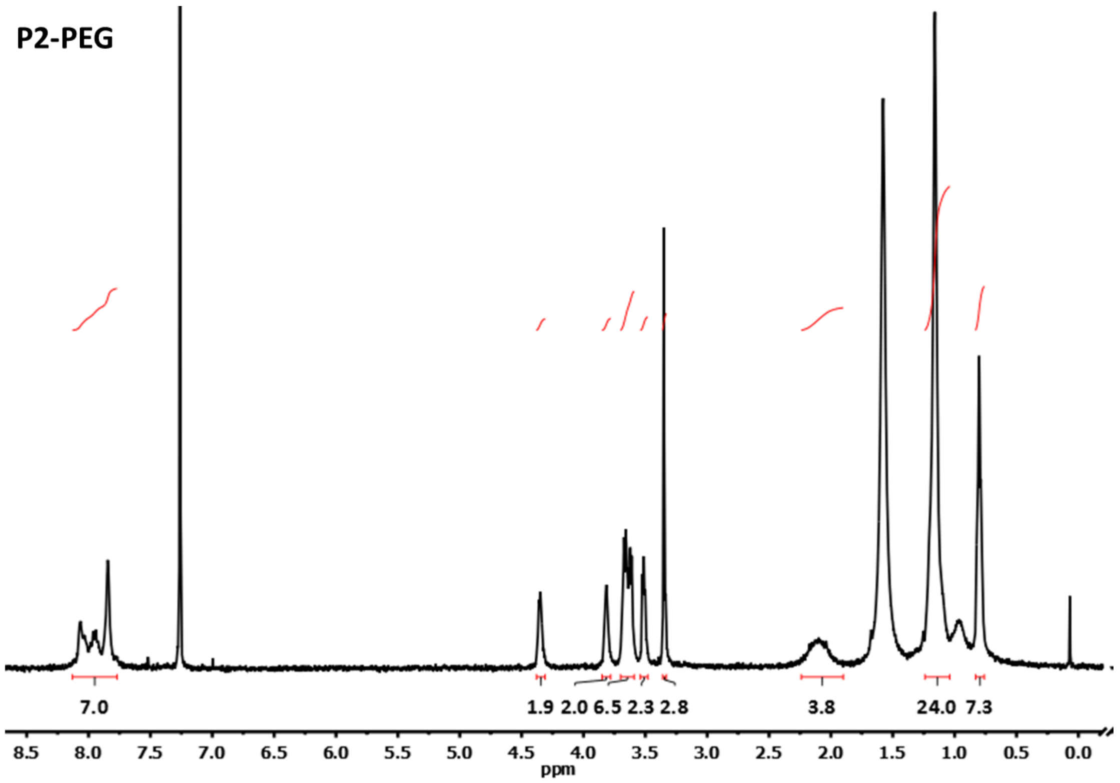

Supplementary Figure 14: ^{1}H NMR of P2-PEG. ^{1}H NMR of polymer **P2-PEG** in $CDCl_3$ at 400 MHz.

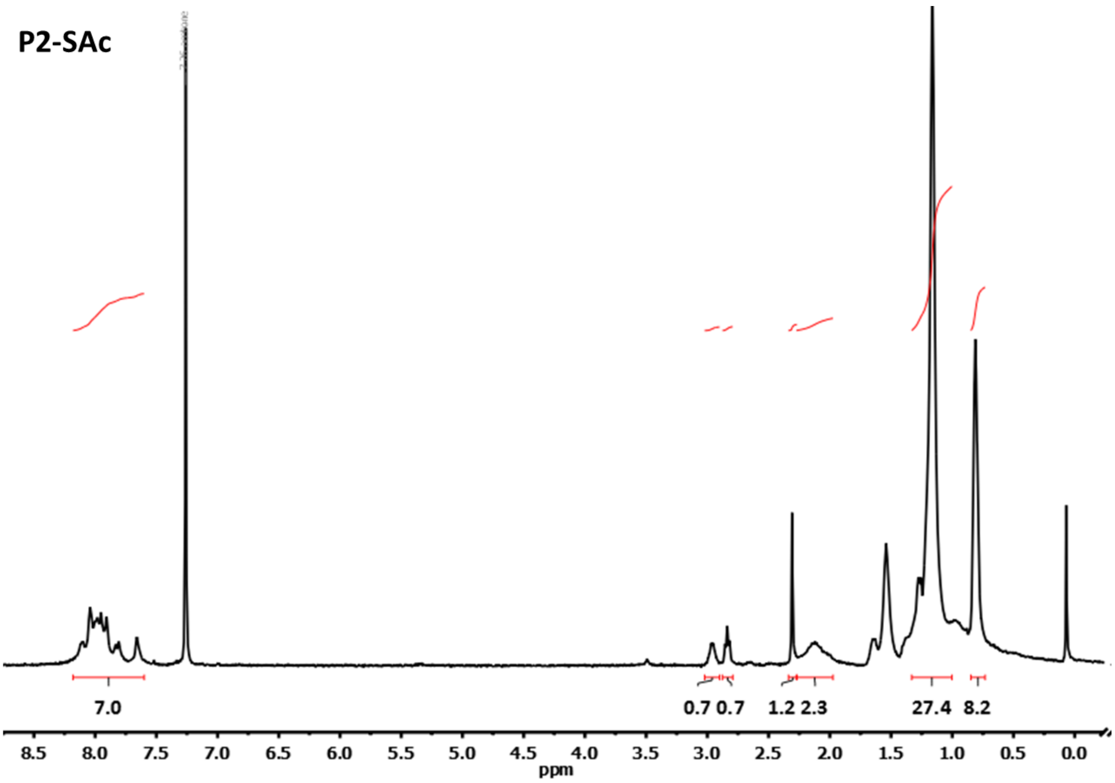

Supplementary Figure 15: ^{1}H NMR of P2-SAc. ^{1}H NMR of polymers **P2-SAc** in $CDCl_3$ at 400 MHz.

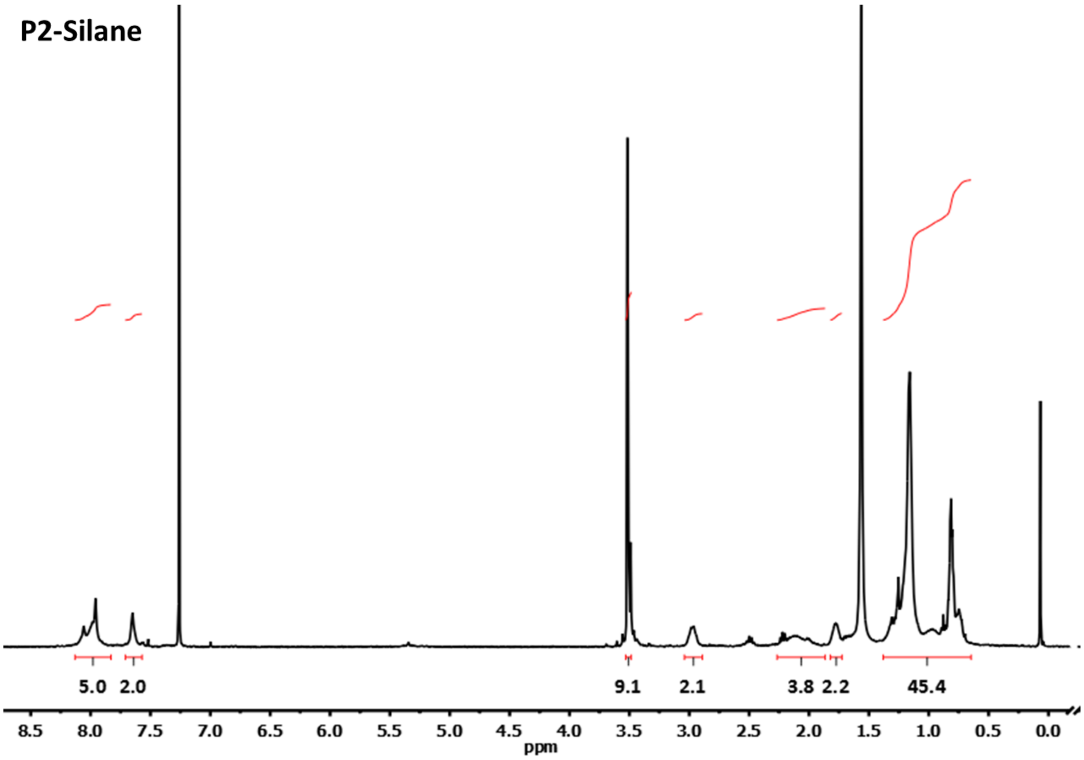

Supplementary Figure 16: ^{1}H NMR of P2-Silane. ^{1}H NMR of polymers **P2-Silane** in $CDCl_3$ at 400 MHz.

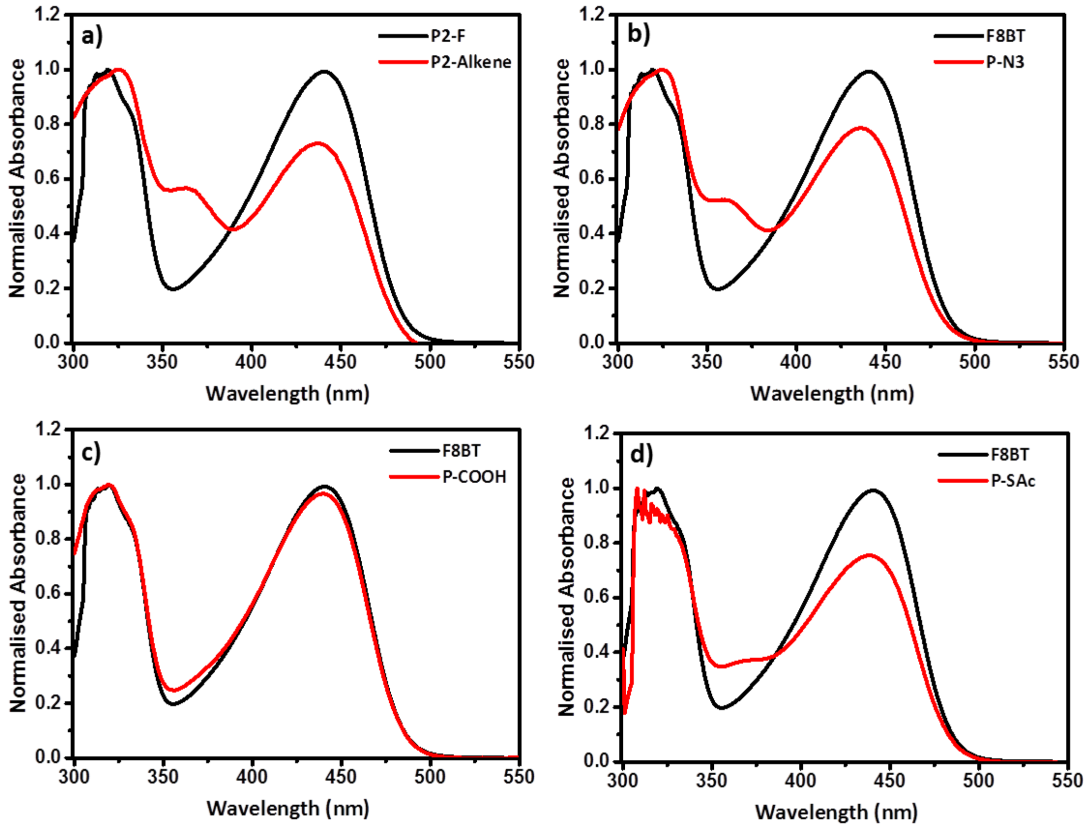

Supplementary Figure 17: Normalised UV-Vis absorption spectra of P2-based polymers. Normalised UV-Vis absorption spectra of a) **P2-Alkene**, b) **P2-N_3**, c) **P2-COOH** and d) **P2-SAc** in chlorobenzene.

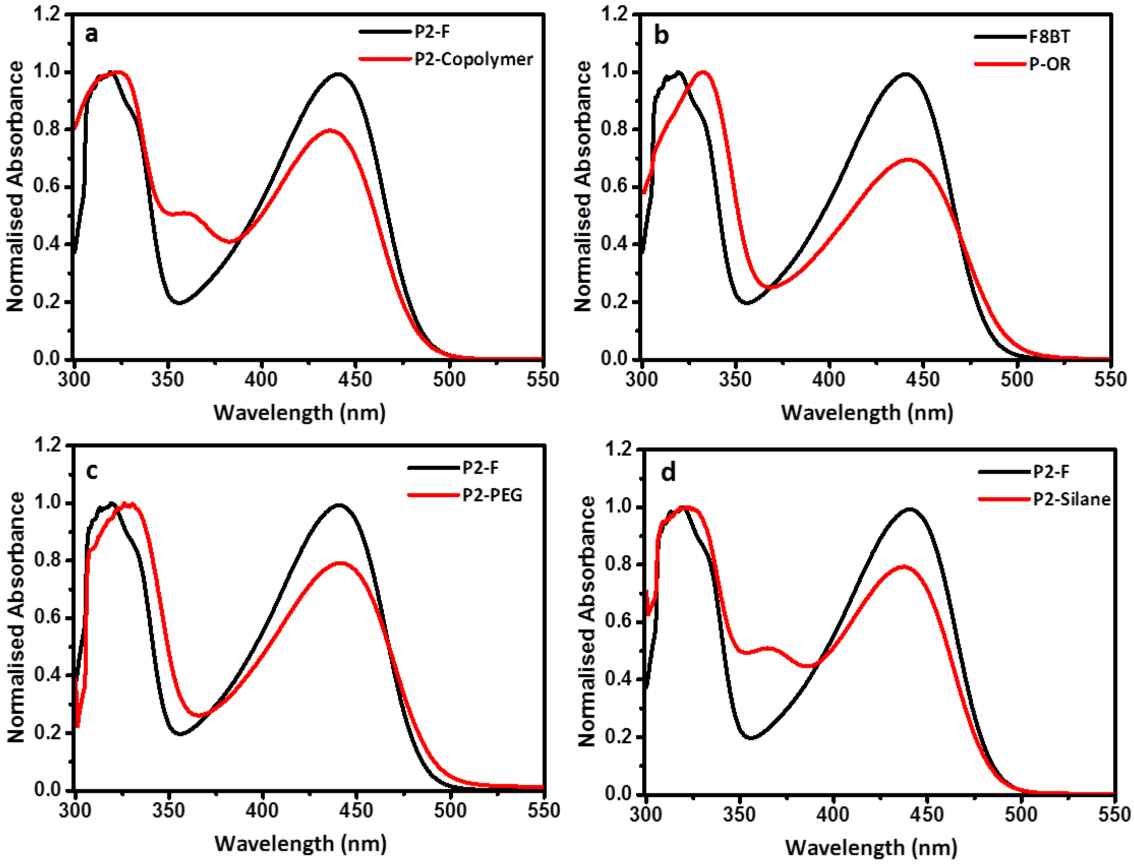

Supplementary Figure 18: Normalised UV-Vis absorption spectra of P2-based polymers. Normalised UV-Vis absorption spectra of a) **P2-Copolymer**, b) **P2-OR**, c) **P2-PEG** and d) **P2-Silane** in chlorobenzene.

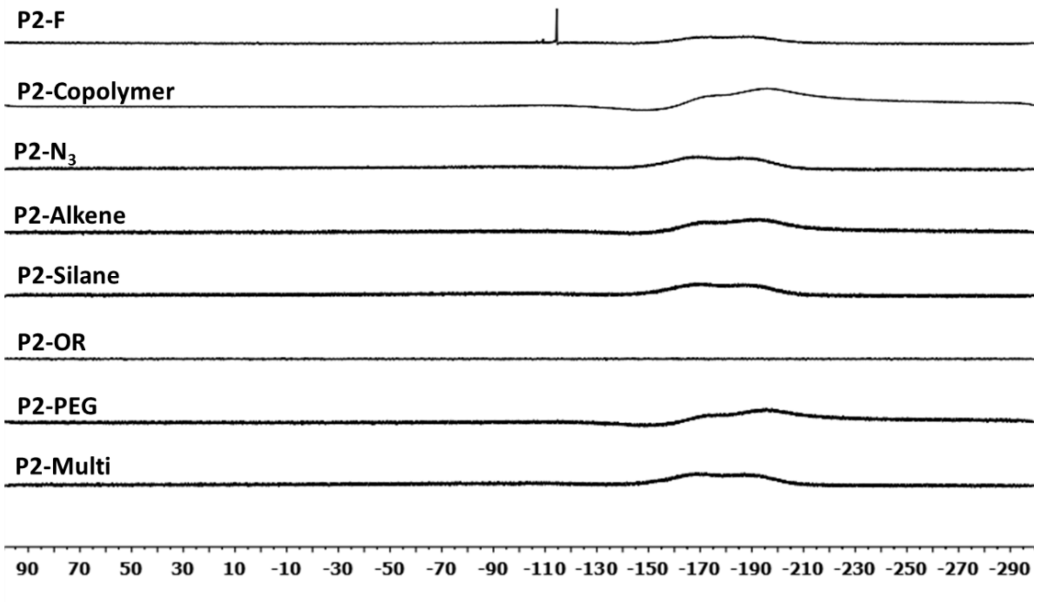

Supplementary Figure 19: ^{19}F NMR of P2-based polymers. ^{19}F NMR of **P2-F, P2-Copolymer, P2-N_3, P2-Alkene, P2-Silane, P2-OR, P2-PEG, P2-Multi** in $CDCl_3$ at 400 MHz.

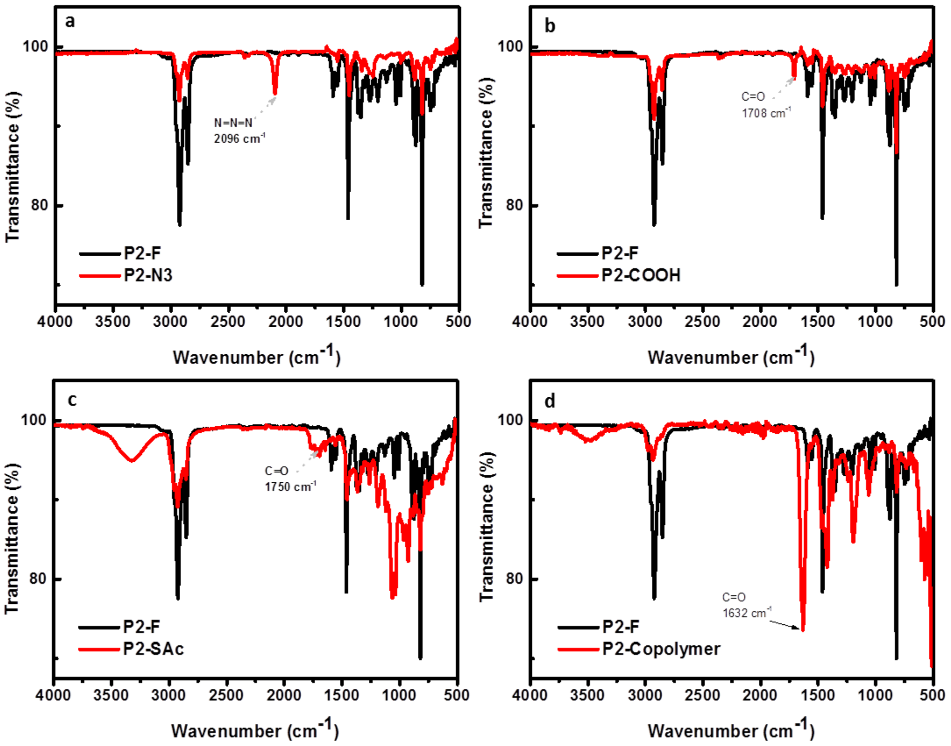

Supplementary Figure 20: ATR-IR of P2-based polymers. ATR of a) **P2-N_3**, b) **P2-COOH**, c) **P2-SAc** and d) **P2-Copolymer** all compared to **P2-F**, in solid phase.

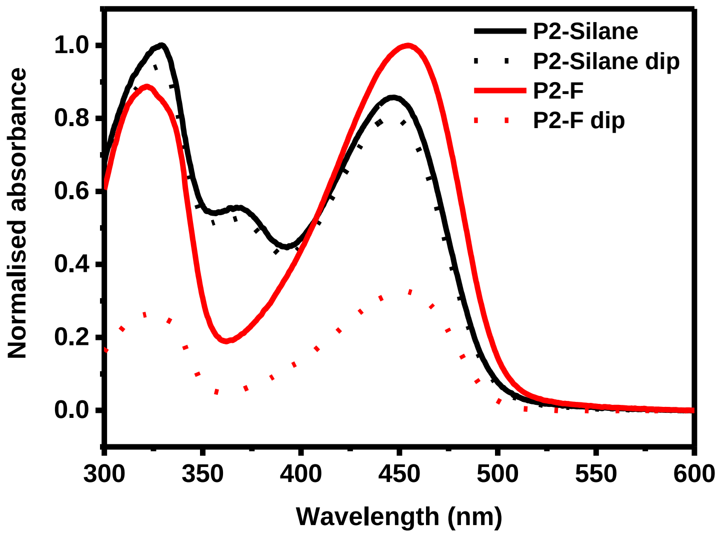

Supplementary Figure 21: Normalised UV-Vis spectra of thin films of P2-Silane and P2-F before and after submersing in $CHCl_3$. Spectra of **P2-Silane** (black lines) and **P2-F** (red lines) before and after submersion in $CHCl_3$ (solid and dashed lines respectively). Spectra normalised with respect to the spectra recorded prior to submersion.

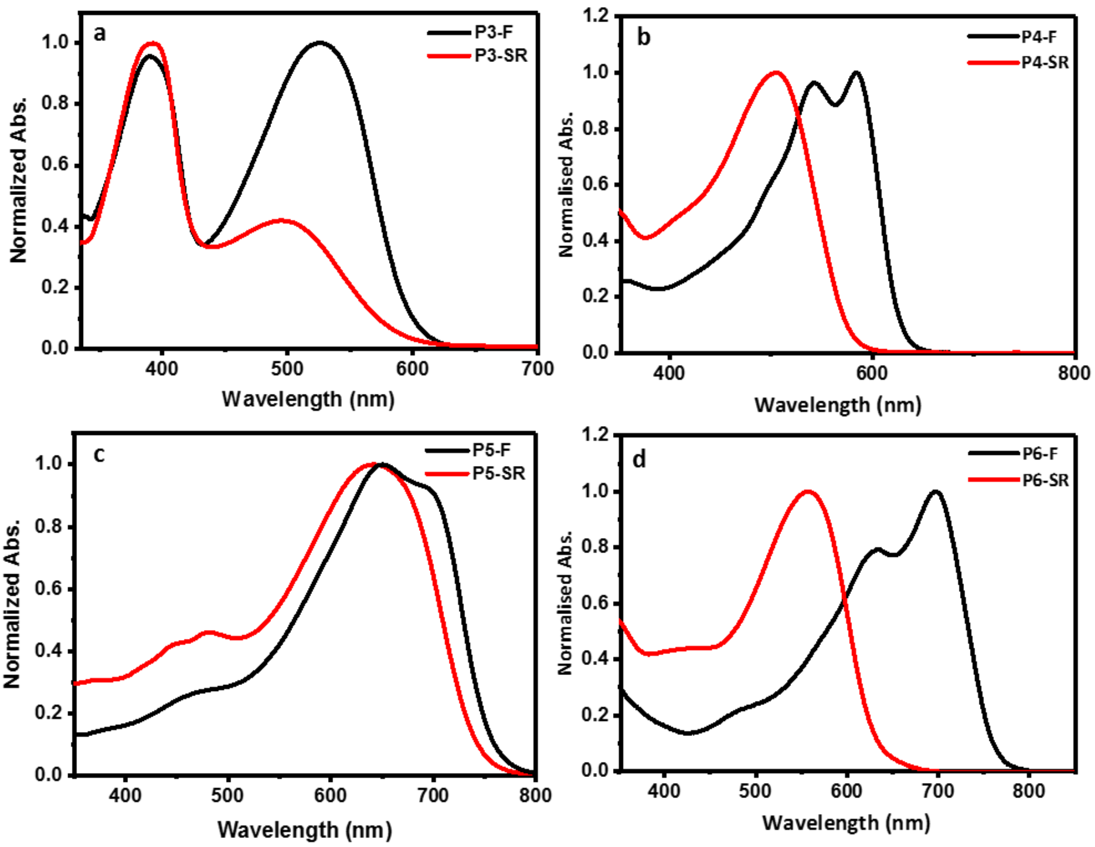

Supplementary Figure 22: Normalised UV-Vis spectra of polymers P3, P4, P5 and P6 before and after substitution reaction. Normalised spectra of a) **P3-F** and **P3-SR**, b) **P4-F** and **P4-SR**, c) **P5-F** and **P5-SR** and d) **P6-F** and **P6-SR** in chlorobenzene at room temperature.

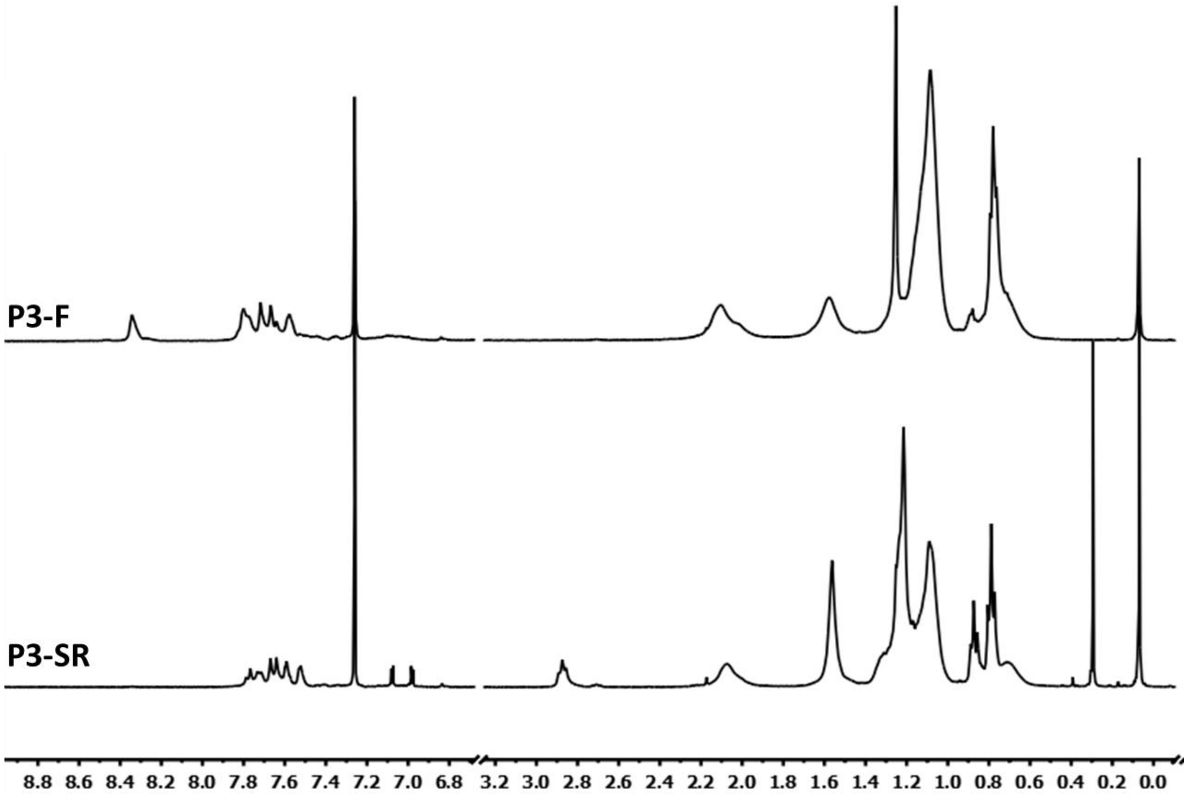

Supplementary Figure 23: ^{1}H NMR of polymer P3 before and after substitution reaction. ^{1}H NMR of **P3-F** and **P3-SR** in $CDCl_3$ at 400 MHz.

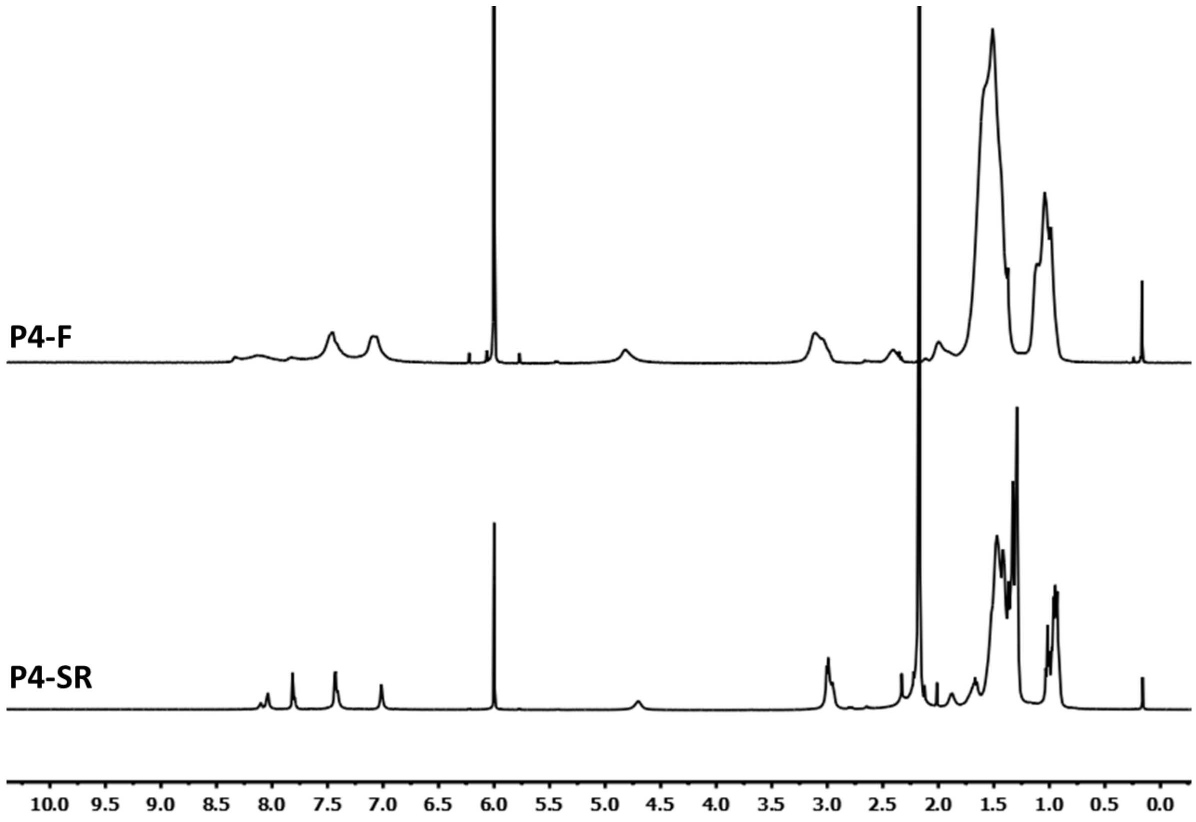

Supplementary Figure 24: ^{1}H NMR of polymer P4 before and after substitution reaction. P4-F and **P4-SR** in TCE-d2 (at 403K) at 400 MHz.

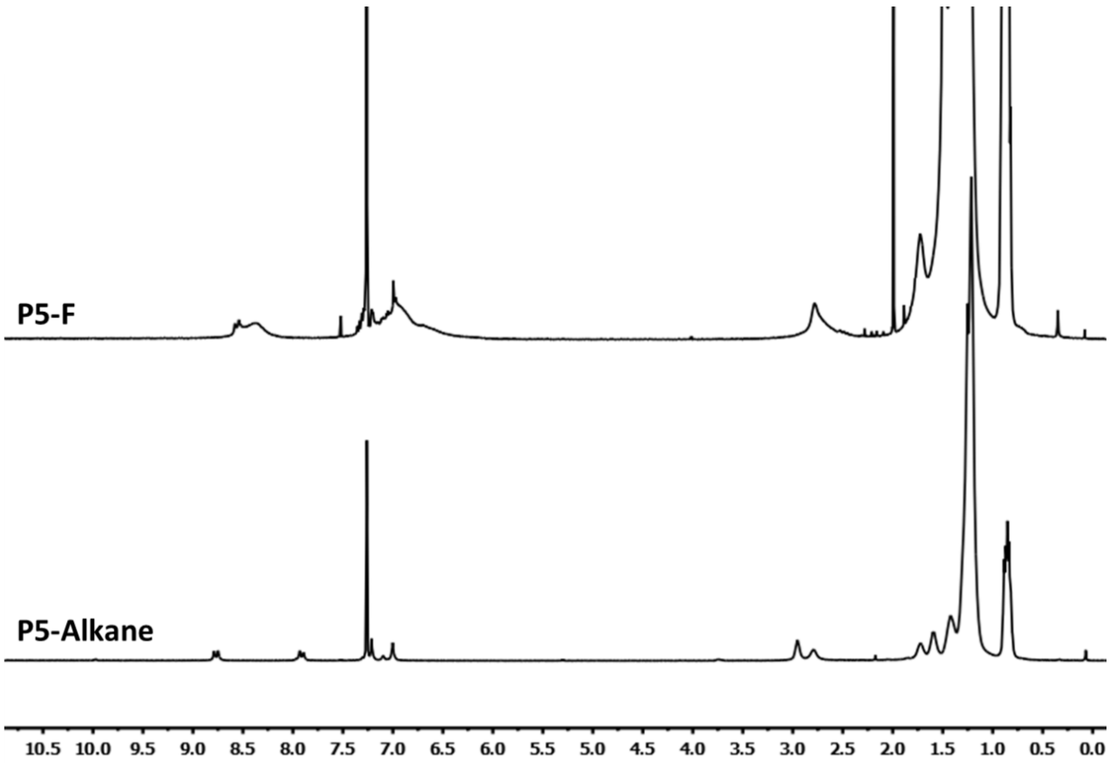

Supplementary Figure 25: ^{1}H NMR of polymer P5 before and after substitution reaction. P5-F and **P5-SR** in $CDCl_3$ at 400 MHz.

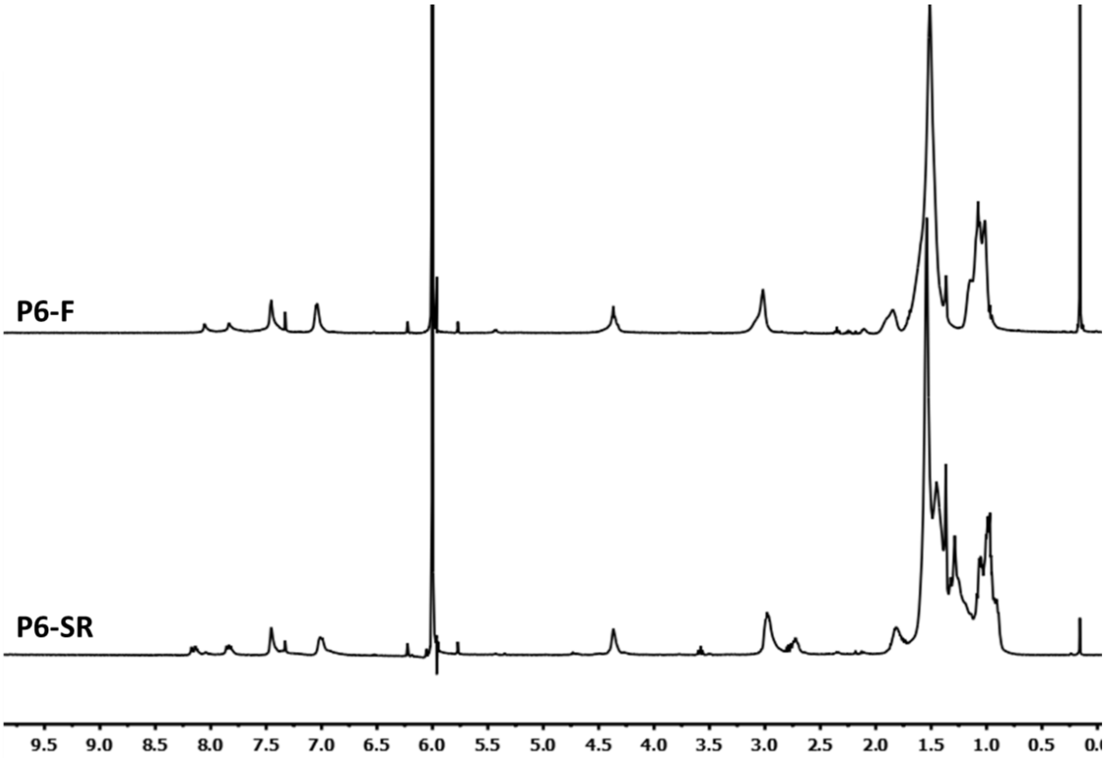

Supplementary Figure 26: ^{1}H NMR of polymer P6 before and after substitution reaction. P6-F and **P6-SR** in TCE-d2 (at 403K) at 400 MHz.

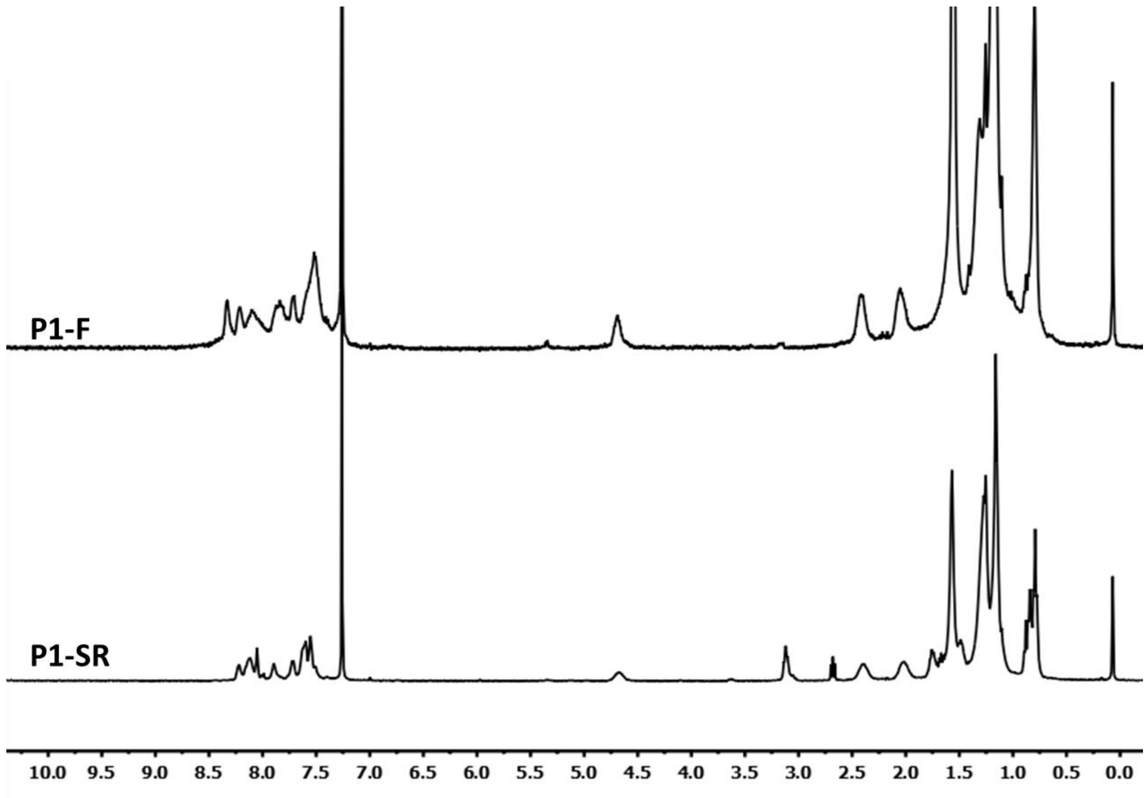

Supplementary Figure 27: ^{1}H NMR of polymer P1 before and after substitution reaction. P1-F and **P1-SR** in $CDCl_3$ at 400 MHz.

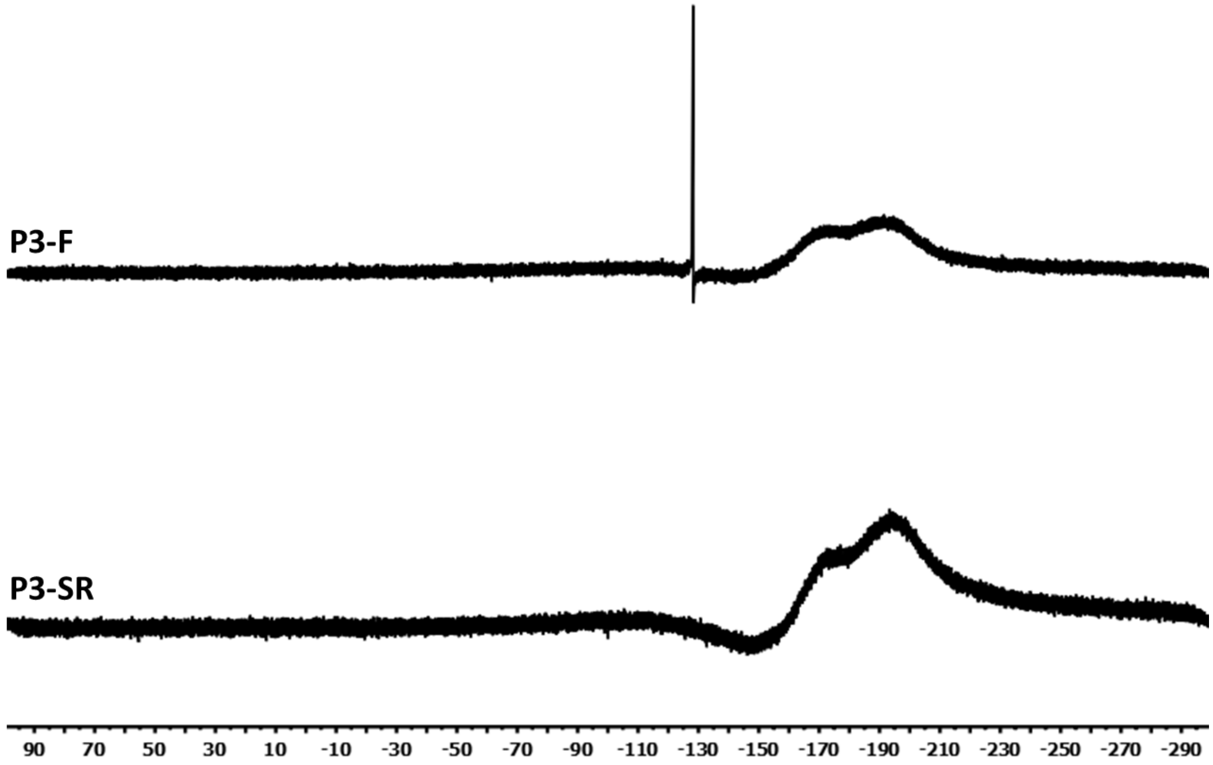

Supplementary Figure 28: ^{19}F NMR of polymer P3 before and after substitution reaction. ^{19}F NMR of **P3-F** and **P3-SR** in $CDCl_3$ at 400 MHz.

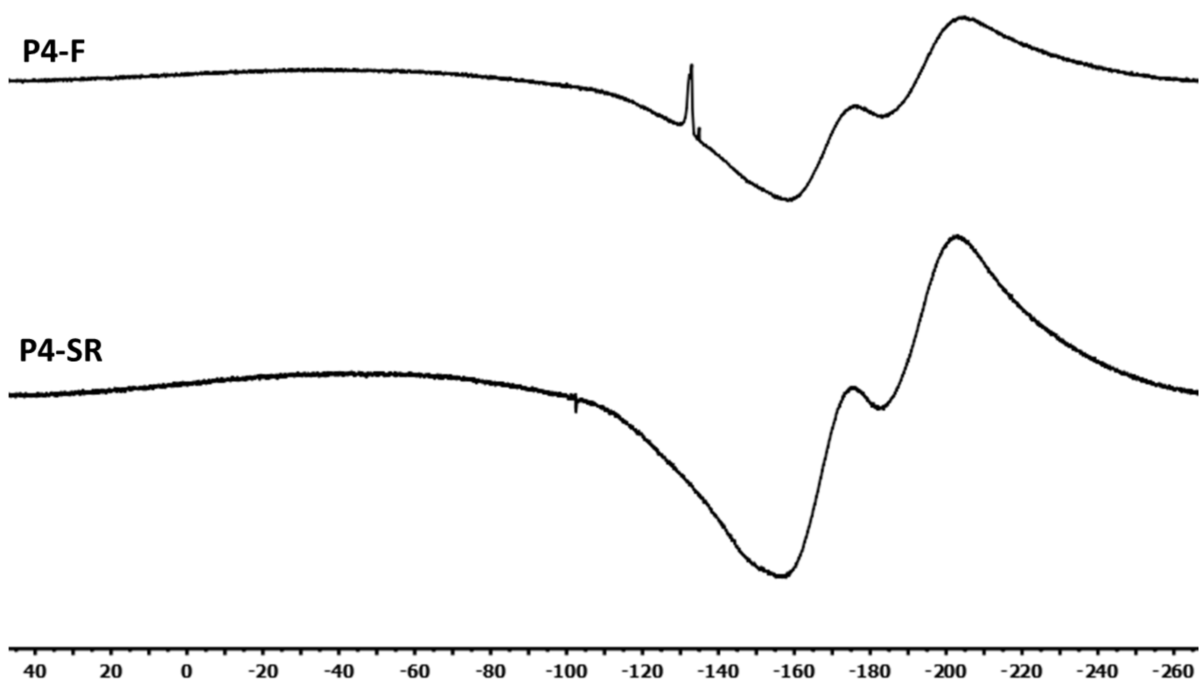

Supplementary Figure 29: ^{19}F NMR of polymer P4 before and after substitution reaction. ^{19}F NMR of **P4-F** and **P4-SR** in TCE-d2 (at 403K) at 400 MHz.

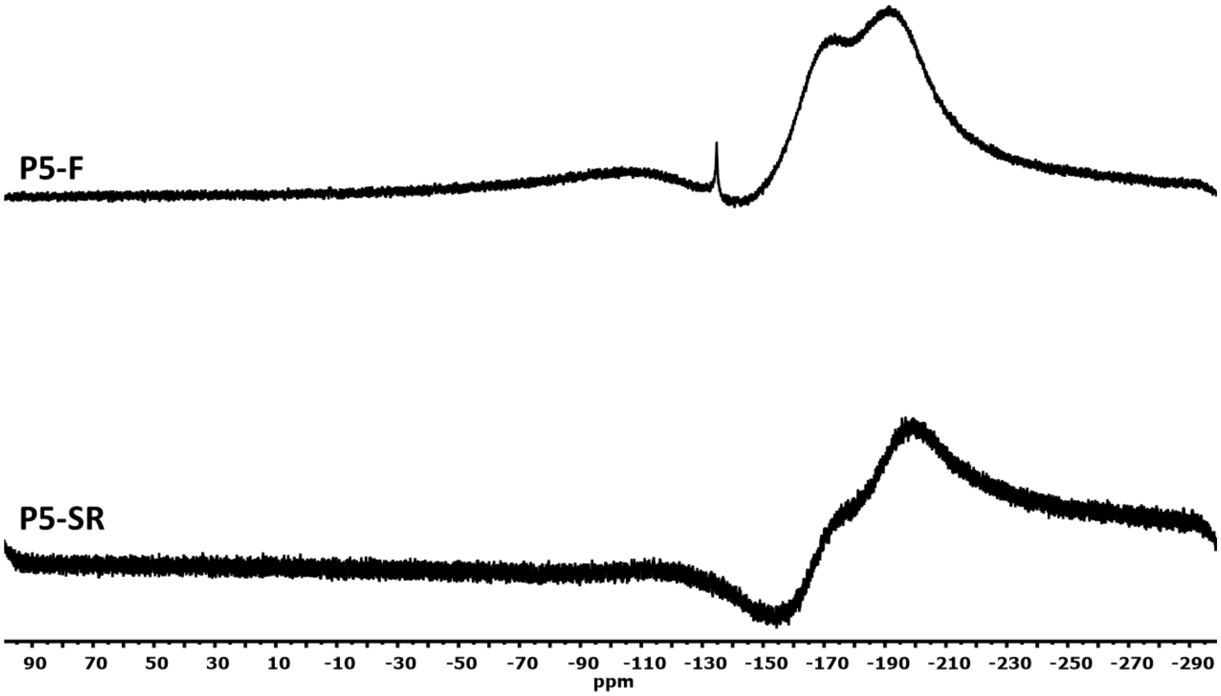

Supplementary Figure 30: ^{19}F NMR of polymer P5 before and after substitution reaction. ^{19}F NMR **P5-F** and **P5-SR** in $CDCl_3$ at 400 MHz.

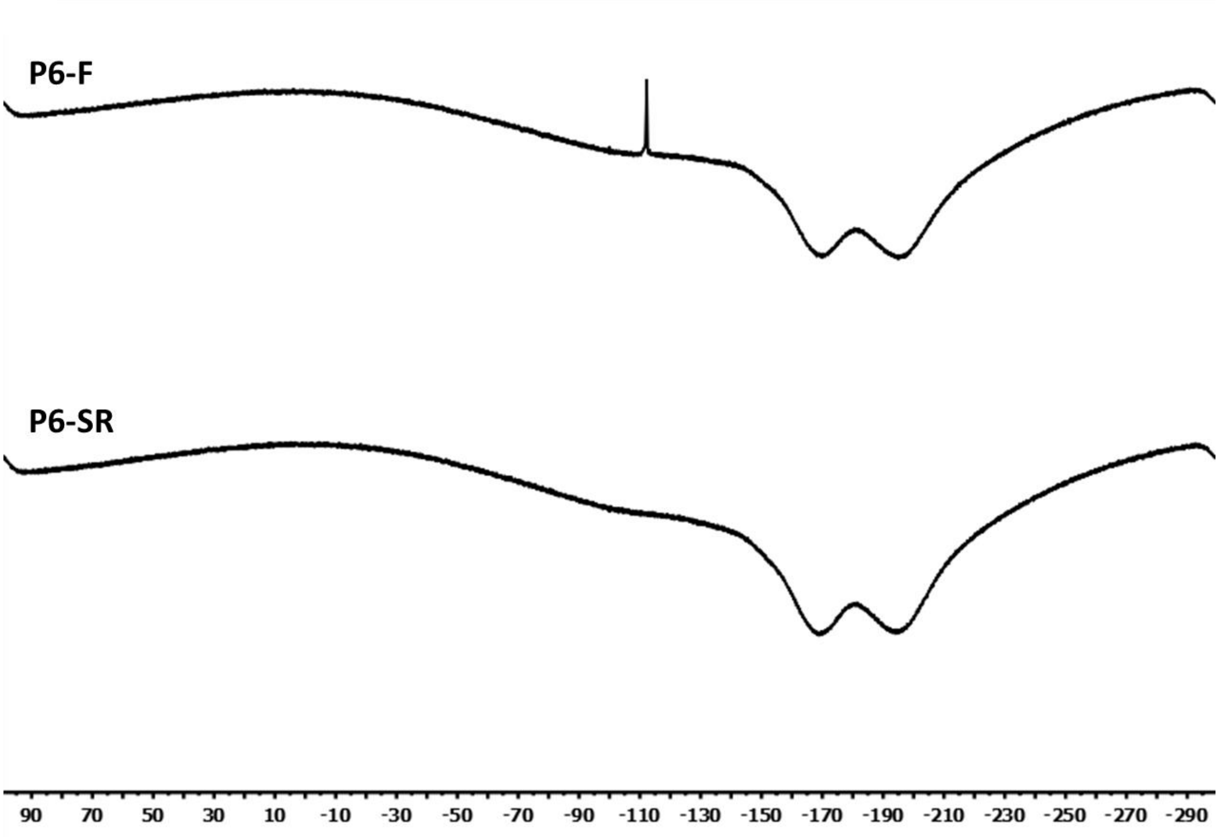

Supplementary Figure 31: ^{19}F NMR of polymer P6 before and after substitution reaction. ^{19}F NMR **P6-F** and **P-6-SR** in TCE-d2 (at 403K) at 400 MHz.

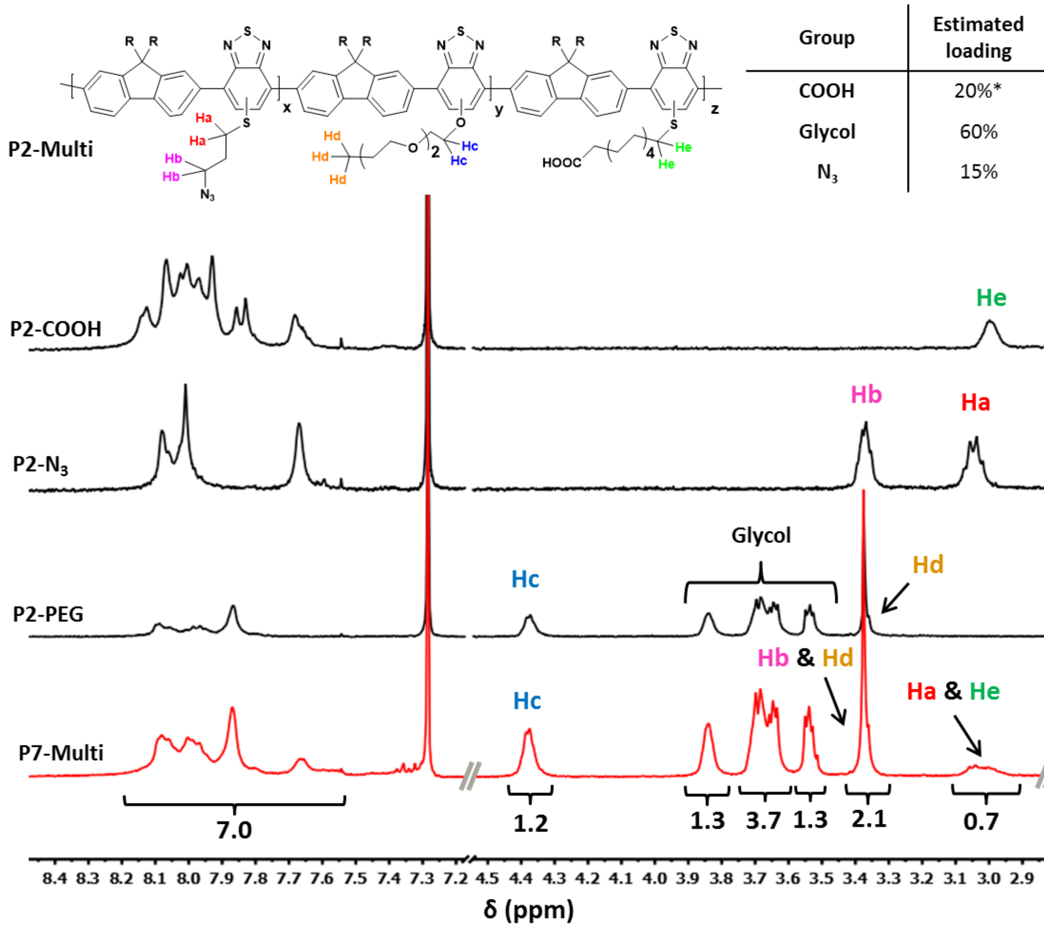

Group	Estimated loading
COOH	20%*
Glycol	60%
N_3	15%

Supplementary Figure 32: ^{1}H NMR spectrum and structure of P2-Multi. Key proton peaks labelled with integration values. Includes NMR spectra of **P2-COOH**, **P2-N_3** and **P2-PEG** for reference.

*Loading of carboxylic acid estimated after initial synthesis of carboxylic acid functionalised polymer.

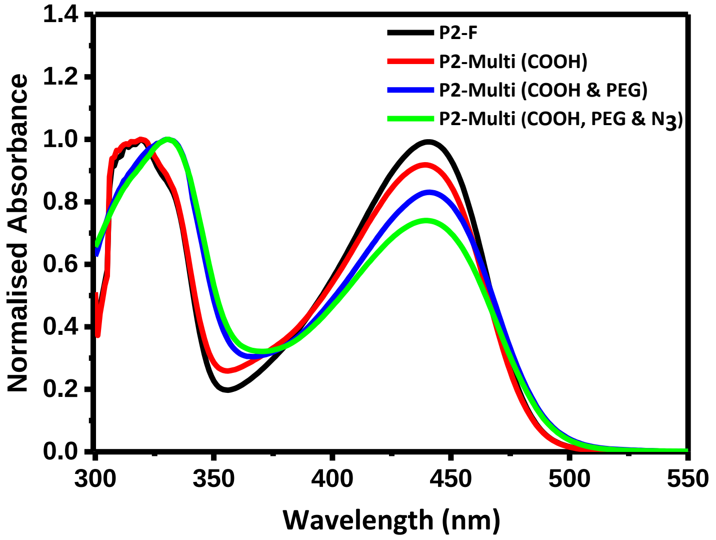

Supplementary Figure 33: Normalised UV-Vis spectra tracking the synthesis of P2-Multi from initial polymer P2-F. Normalised UV-Vis spectra of **P2-F** (black line) after reaction with: 25 mol% of 11-mercaptoundecanoic acid (red line) followed by 60 mol% triethylene glycol monomethyl ether (blue line) and excess *S*-(3-azidopropyl)thioacetate (green line) to synthesise **P2-Multi**.

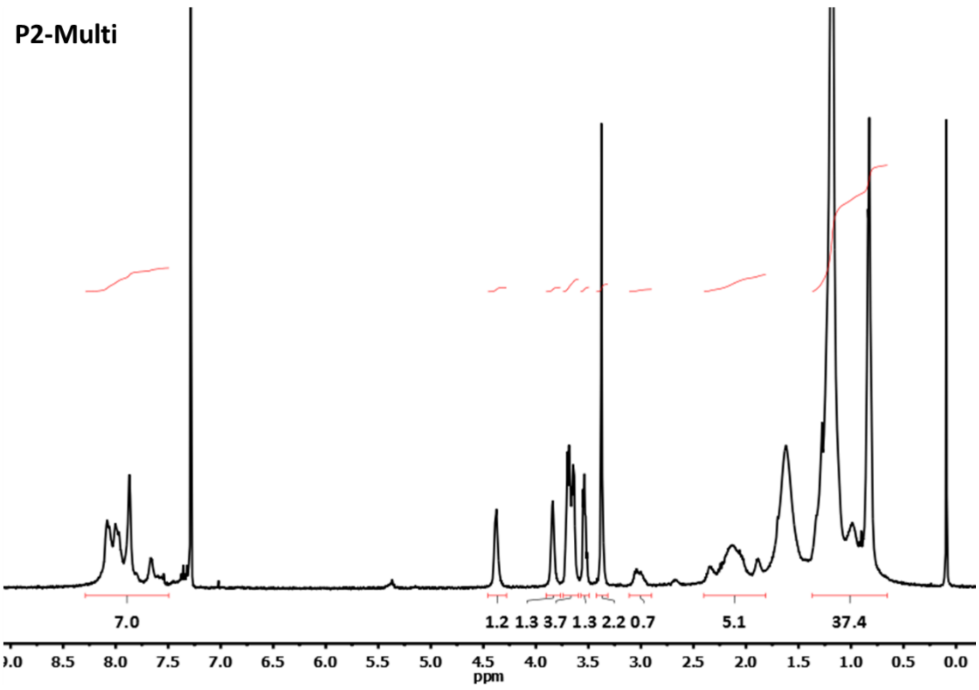

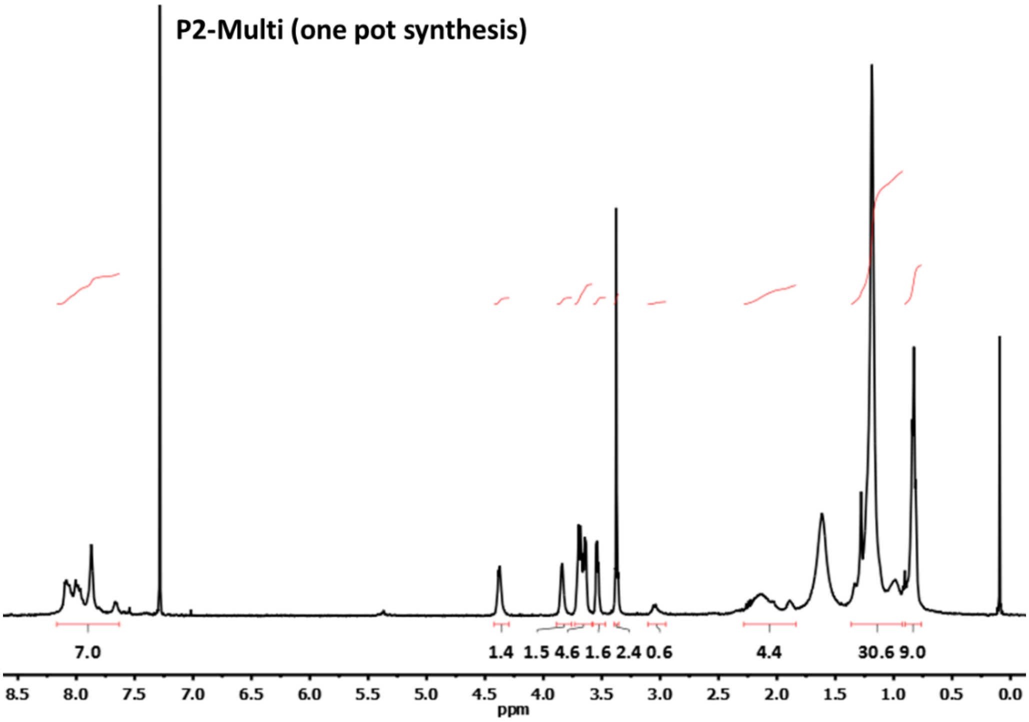

Supplementary Figure 34: ^{1}H NMR of P2-Multi from multi step and one pot synthesis. ^{1}H NMR of P2-Multi and P2-Multi (one pot synthesis) in $CDCl_3$ at 400 MHz.

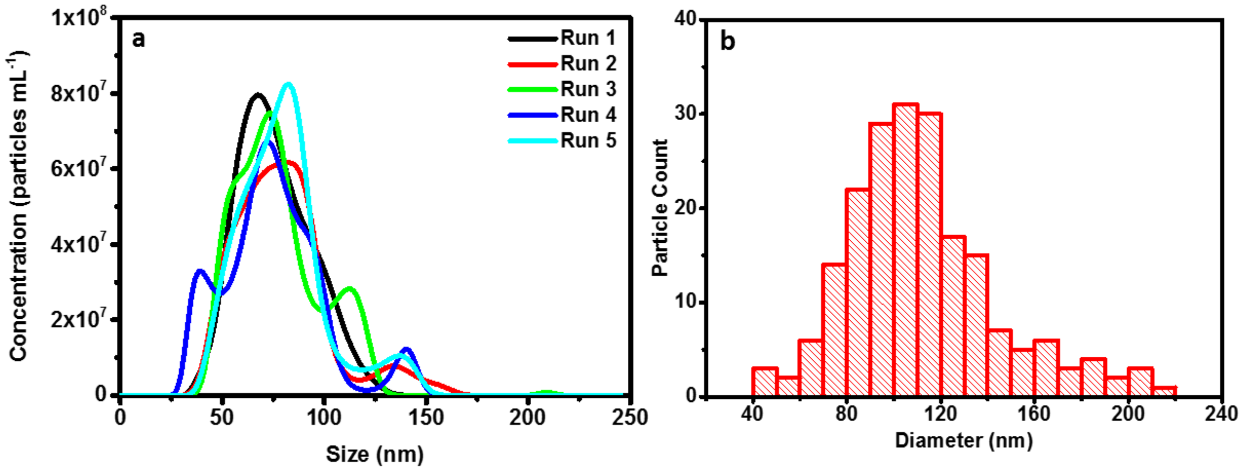

Supplementary Figure 35: Size distributions of SPN-N_3 nanoparticles in water. a) calculated using nanoparticle tracking analysis (NTA) (5 separate videos analysed) and b) scanning transmission electron microscopy (STEM) (100 particles manually counted).

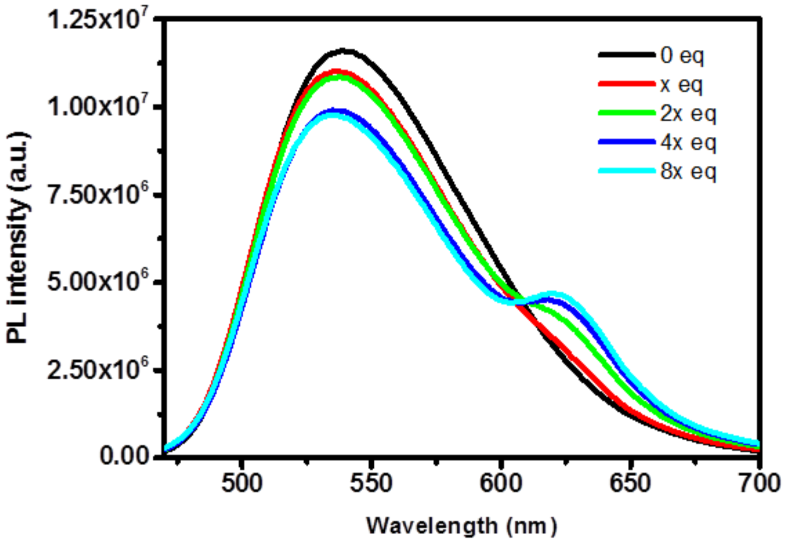

Supplementary Figure 36: PL emission spectra of SPN- N_3 with increasing equivalents of inactive active dye. x = 423 dye molecules per nanoparticle. Samples were pulsed at 450 nm, all at the same nanoparticle concentration (concentration of nanoparticle calculated with NTA).

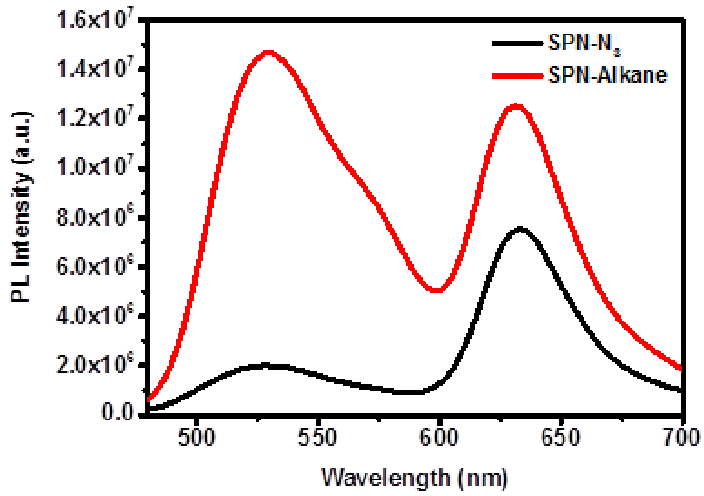

Supplementary Figure 37: PL spectra of azide and octyl functionalised nanoparticles with an excess of reactive dye. Spectra of **SPN-N_3** (red line) and **SPN-SR** (black line) particles with excess reactive dye.

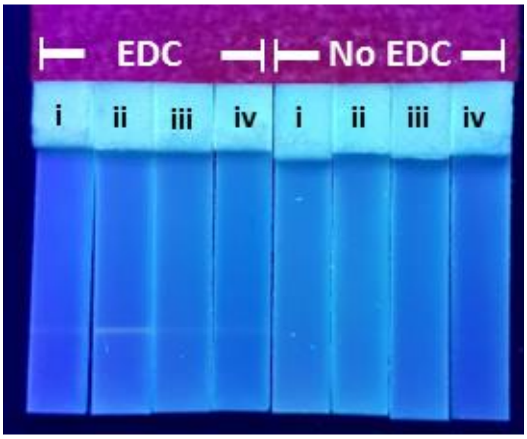

Supplementary Figure 38: Photo of nitrocellulose based lateral flow strips with SPN-Multi nanoparticles. Strips functionalised with a poly-streptavidin test line 4 cm from the base, under UV exposure (365 nm). Before running samples up each strip, **SPN-Multi** nanoparticles were reacted with i) 1, ii) 10, iii) 100 and iv) 200 µg/mL solutions of biotin-NH_2 (O-(2-Aminoethyl)-O'-[2-(biotinylamino)ethyl]octaethylene glycol) with and without EDC present. The reaction solutions were then run up the strips and allowed to dry. We speculate that the reduction in fluorescence intensity at the test line for concentrations of biotin over 10 ul is caused by unreacted biotin in solution. This can outcompete the particles when binding at the test line.

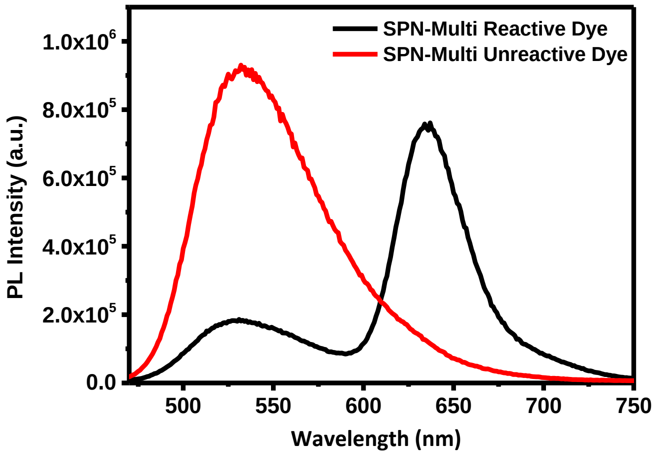

Supplementary Figure 39: PL spectra of the SPN-Multi solutions with reactive and unreactive dye. SPN-Multi with reactive dye (black line) and unreactive dye (red line). Solutions excited at 450 nm with 10 vol% THF added.

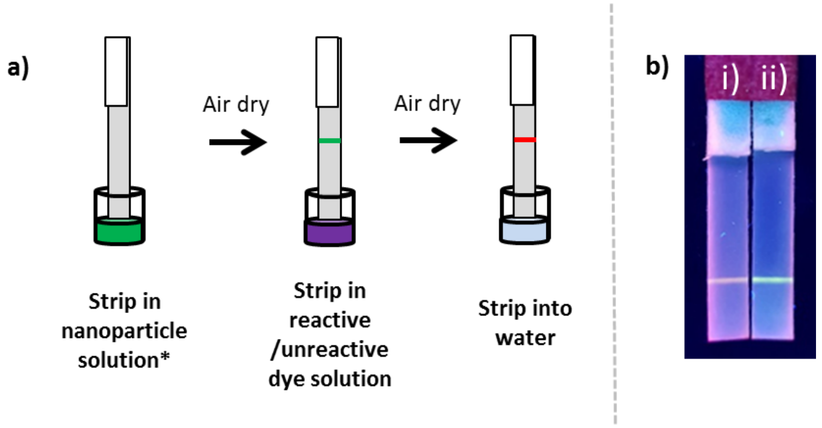

Supplementary Figure 40: Click reaction directly onto nitrocellulose strips. a) Schematic of click reaction directly on test line of nitrocellulose strips with b) image of strips reacted with i) reactive and ii) unreactive MB-594 dye (irradiated with UV light (365 nm). *solutions are incubated in fetal bovine solution (FBS) with TWEEN®20 (0.02 vol%) prior to the adding of the strip.

Supplementary Discussion

Calculation of FRET efficiency from PL and TCSPS data on SPN-N$_3$

FRET efficiencies (E_{FRET}) were calculated from the PL spectra (Figure 5c (main article) and Supplementary Figure 36 for active and inactive dye, respectively) using Supplementary Equation 1.

$$E_{FRET} = 1 - \frac{I_{DA}}{I_D} \tag{1}$$

Where *I* refers to the maximum fluorescence intensity of the donor in the presence (DA) or absence (D) of acceptor. Summary of the FRET efficiencies calculated from PL spectra can be found in Supplementary Figure 45.

To provide further evidence of that a FRET mechanism was occurring, the lifetime of the excited state (of the donor) with increasing acceptor equivalents was recorded using time-correlated single photon counting (TCSPC). The fluorescence decay of the free **SPN-N$_3$** is complex in itself. The decay shows some curvature in a log-lin plot, indicating that a single exponential decay is an inappropriate model. Good fits (Supplementary Figure 41) were obtained using a stretched exponential model[1] (Supplementary Equation 2).

$$I(t) = I_0 + A\exp\left[-\left(\frac{x}{t}\right)^{\beta}\right] \tag{2}$$

Where I_0 is the background level, x is the time constant, t is time, and β is the heterogeneity factor. β takes values between 0 and 1, where values closer to 1 indicate a system with less heterogeneity. The average lifetime is calculated using Supplementary Equation 3.

$$\langle\tau\rangle = \beta x \Gamma(\beta) \tag{3}$$

Where Γ is the mathematical gamma function.

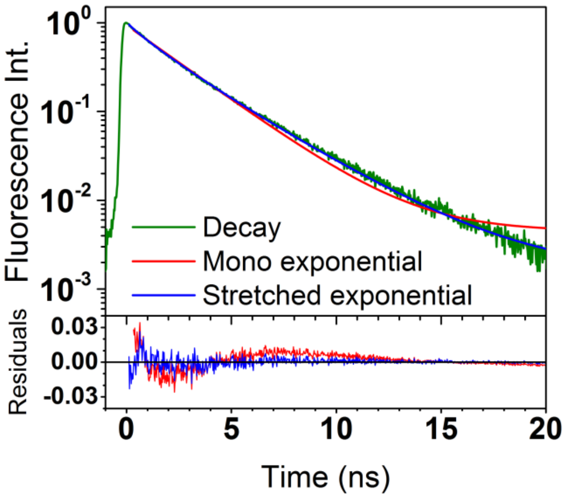

Supplementary Figure 41: Fits of the time-resolved fluorescence decay of free SNP-N_3. A mono exponential fit is compared to a stretched exponential fit. Fit residuals are shown below.

The heterogeneity factor (β = 0.87) has a value near 1, consistent with small degree of heterogeneity possibly due to a distribution of nanoparticle size or polymer chain conformation. The average lifetime of free **SPN-N_3** obtained from the stretched exponential fit is 2.44 ns.

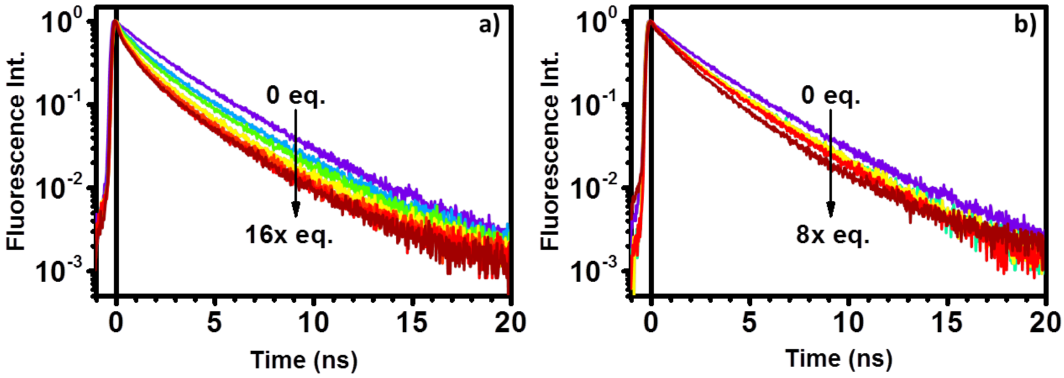

Supplementary Figure 42: Time-Correlated Single Photon Counting (TCSPC) decay trace of SPN-N_3 nanoparticles. SPN-N_3 with up to a) 16x eq. of reactive and up to b) 8x eq. of unreactive dye. Here x = 423.

The decay traces of the reaction solutions, with increasing reactive and unreactive dye amounts, were measured. The decay traces of **SPN-N_3** with increasing equivalents of reactive and unreactive dye can be found in Supplementary Figure 42a and b, respectively. It can be seen that the excited state decays faster in the presence of the reactive dye, compared to the unreactive dye analogue.

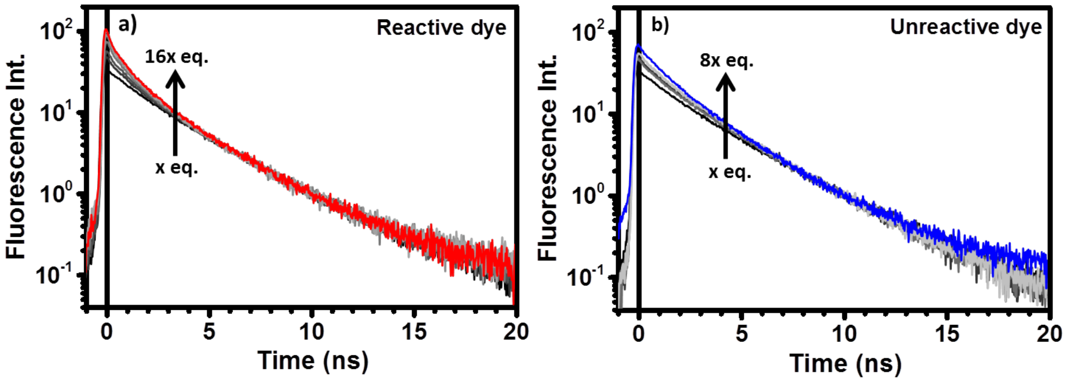

Supplementary Figure 43: TCPSPC decays of SPN-N$_3$ with reactive and unreactive dye. **SPN-N$_3$** with **a)** reactive dye and **b)** unreactive dye, normalised at 10 ns (x = 423).

It can also be seen that the decay traces of the nanoparticles with dye converge with those of the free nanoparticle at times approximately longer than 10 ns (Supplementary Figure 42). This is illustrated for all equivalents of dye by the plot of decay traces normalised at 10 ns, where all systems showed similar decays regardless of dye concentration beyond 10 ns (Supplementary Figure 43). As a result, the FRET component could be extracted by subtracting the free nanoparticle trace from each decay trace and isolating the first 5 ns of decay. The resulting decays (normalised at t = 0) can be found in Supplementary Figure 44. The shape of the decay traces can be seen to be independent of dye equivalents and only dependent on the type of dye used (reactive or unreactive). The FRET efficiency of the extracted data was calculated using a stretched exponential fit of each of the decay traces. From the extracted FRET decays, we may extract $<k_{FRET}>$ from the average FRET lifetimes obtained by a stretched exponential fit. For the reactive acceptors $<k_{FRET}>$ = $1.6 \pm 0.2 \times 10^9$ s^{-1} and for the unreactive $<k_{FRET}>$ = $8.7 \pm 0.3 \times 10^8$ s^{-1}. Considering the average lifetime of free NPs, under kinetic competition (see equation below) we calculate a maximal FRET efficiency value of 0.80 ± 0.02 for the active dye and 0.68 ± 0.01 for the inactive dye (dotted lines in Supplementary Figure 45). The faster FRET rate constant and higher maximal FRET efficiency observed for the reactive dye highlights the success of the click reaction and intimate interaction through formation of a covalent linkage.

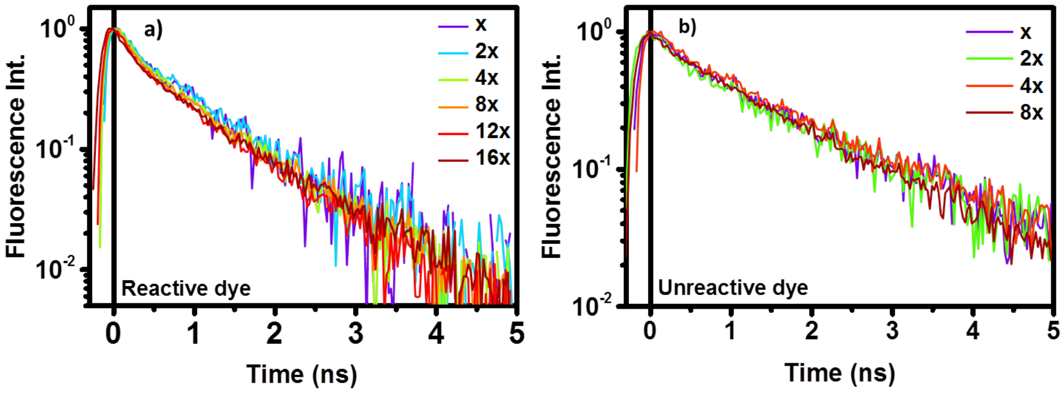

Supplementary Figure 44: Extracted decays of the FRET component. SPN-N₃ reactions with **a)** reactive dye and **b)** unreactive dye at different equivalents of dye (x = 423).

The obtained lifetimes were then used to calculate the FRET efficiency using a similar equation to that used for PL (Supplementary Equation 4).

$$E_{FRET} = 1 - \frac{\tau_{DA}}{\tau_D} \quad (4)$$

Here τ_{DA} and τ_D refer to the donor fluorescence lifetime in the presence or absence of the acceptor, respectively. The FRET efficiencies as a function of dye equivalents are plotted in Supplementary Figure 45. It can be clearly seen that the efficiencies start to plateau at $8x$ equivalents of reactive dye and the levels of efficiency are much lower with unreactive dye. E_{FRET} does not rival the maximal FRET efficiencies, especially in the unreactive case, because the broadly distributed decay processes are competing with the FRET.

The trends observed in E_{FRET} with increasing acceptor concentration, calculated from PL and TCSPC data, also show excellent agreement. Note that there is some level of discrepancy between the photoluminescence and time-resolved measurements. This is most likely due to the simplification in calculating the average lifetime from the stretched exponential fit. Nevertheless, substantial differences are seen between the reactive and unreactive dye reactions, consistent with the formation of a covalent linkage.

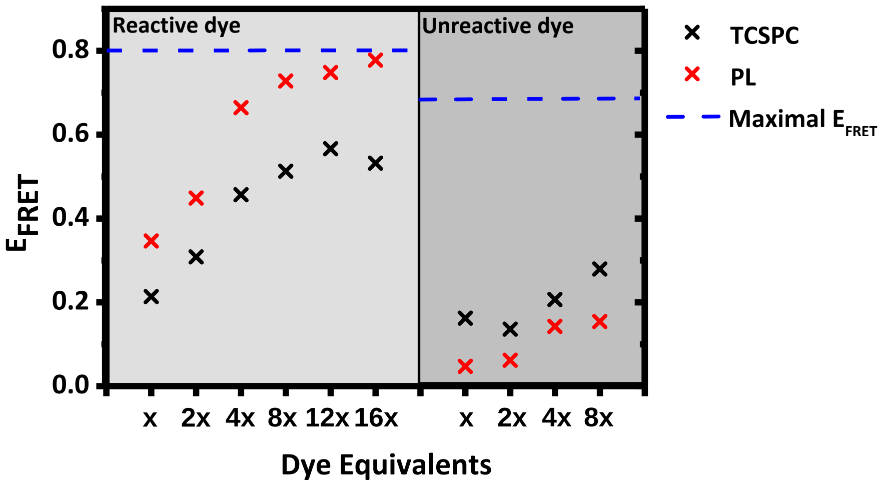

Supplementary Figure 45: FRET efficiency (E_{FRET}) as a function of equivalents of active and inactive dye. Efficiency calculated from PL spectra (red) and time-correlated single photon counting (TCSPC) (black) (x = 423).

Supplementary Methods

Precursor monomers of polymers P1-P6

The precursor materials for polymers **P1-F** to **P5-F** were all either purchased from commercial sources or synthesised according to literature procedure, which will be summarised for each polymer below (**P6-F** was purchased from Ossila):

P1-F - (4,7-Bis(5-bromothiophen-2-yl)-5-fluoro-2,1,3-benzothiadiazole) was synthesised according to literature procedure[1]. 9-(9-heptadecanyl)-9H-carbazole-2,7-diboronic acid bis(pinacol) ester was purchased from Derthon.

P2-F - 4,7-Dibromo-5-fluoro-2,1,3-benzothiadiazole was purchased from Novachemistry, 9,9-dioctyl-9*H*-fluorene-2,7-diboronic acid bis(pinacol) ester was purchased from Sigma Aldrich.

P3-F - 6,6,12,12-Tetraoctyl-6,12-dihydroindeno[1,2-b]fluoren-8-yl]-1,3,2-dioxaborolane diboronic ester was purchased from SunaTec Inc.. The precursor to 4,7-bis(5-bromothiophen-2-yl)-5,6-difluoro-2,1,3-benzothiadiazole, (5,6-difluoro-4,7-di(thiophen-2-yl)-2,1,3-benzothiadiazole) was synthesised according to literature procedure[2] which was then subsequently brominated following a further literature procedure[3].

P4-F - 4,7-Bis(5-bromothiophen-2-yl)-2-(2-butyloctyl)-5,6-difluoro-2H-benzo[d][1,2,3]triazole and 2,6-bis(trimethyltin)-4,8-bis(5-(2-butyloctyl)thiophene-2-yl)-benzo[1,2-b;4,5-b']dithiophene were purchased from SunaTec Inc..

P5-F - 4-Bis(2-octyldodecyl)-5,5-bis-(trimethyltin)-dithieno[3,2-b:2,3-d]germole was synthesised according to literature procedure.[4] (4,7-Bis[(E)-2-(5-bromo-3-dodecylthiophen-2-yl)ethenyl]-5,6-difluoro-2,1,3-benzothiadiazole) was synthesised according to literature procedure.[5]

Synthesis of polymers P1-P5

P1-F – *P(CdTfBT)*

(4,7-Bis(5-bromothiophen-2-yl)-5-fluoro-2,1,3-benzothiadiazole) (149.3 mg, 0.315 mmol), 9-(9-heptadecanyl)-9H-carbazole-2,7-diboronic acid bis(pinacol) ester (206.1 mg, 0.315 mmol), $Pd(PPh_3)_4$ (7.2 mg, 0.006 mmol) and a stirrer bar were added to a 5 mL high pressure microwave reactor vial. The vial was then sealed with a septum and flushed with argon, before degassed toluene (3 mL), degassed aqueous 1 M Na_2CO_3 (0.6 mL) and a drop of aliquot-336 were added. The resulting solution was degassed for 30 min before the reaction was heated to 120 °C for 3 days. The reaction was cooled to room temperature, precipitated in methanol (100 mL), stirred for 30 min and filtered through a Soxhlet thimble. The polymer was extracted (Soxhlet) using methanol, acetone, hexane and chloroform in that order under argon. The chloroform fraction was concentrated to ~1 mL before precipitation into methanol (10 mL). The suspension was stirred for 30 min, filtered, redissolved in chloroform and precipitated into methanol again to yield **P1-F** as a black solid (5 mg, 2%). Mn of 3.8 kDa, Mw of 4.9 kDa, Mw/Mn (Đ) = 1.29; ^{1}H NMR (400 MHz, $CDCl_3$) δ 8.28 – 7.43 (m, 11H), 4.81 – 4.55 (br, 1H), 2.50 – 2.27 (br, 2H), 2.11 – 1.96 (br, 2H), 1.42 – 1.07 (m, 22H), 0.90 – 0.74 (m, 6H); ^{19}F NMR (400 MHz, $CDCl_3$) δ -108.1.

P2-F – *P(F8fBT)*

4,7-Dibromo-5-fluoro-2,1,3-benzothiadiazole (290.1 mg, 0.930 mmol), 9,9-dioctyl-9*H*-fluorene-2,7-diboronic acid bis(pinacol) ester (597.7 mg, 0.930 mmol), $Pd(PPh_3)_4$ (21.5 mg, 0.019 mmol) and a stirrer bar were added to a 20 mL high pressure microwave reactor vial. The vial was then sealed with a septum and flushed with argon, before degassed toluene (8 mL), degassed aqueous 2 M Na_2CO_3 (5 mL) and a drop of aliquot-336 was added. The resulting solution was degassed for 30 min before the reaction was heated to 120 °C for 3 days. The reaction was cooled to room temperature, precipitated in methanol (100 mL), stirred for 30 min and filtered through a Soxhlet thimble. The polymer was then extracted (Soxhlet) using methanol, acetone, hexane and chloroform in that order under argon. The chloroform fraction was concentrated to ~10 mL before precipitation into methanol (100 mL). The suspension was stirred for 30 min, filtered, redissolved in chloroform and precipitated into methanol again to yield **P2-F** as a yellow solid (413 mg, 82%); ^{1}H NMR (400 MHz, $CDCl_3$) δ 8.20 – 7.71 (m, 7H), 2.40 – 1.88 (m, 4H), 1.30 – 0.87 (m, 24H), 0.85 – 0.76 (m, 6H); ^{19}F NMR (400 MHz, $CDCl_3$) δ -114.5; Anal. Calcd. for $C_{35}H_{41}FN_2S$ C 77.74, H 7.64, N 5.18, found: C 77.54, H 7.68, N 4.57. Multiple batches synthesised with various molecular weights, batch 1: M_n: 50 kDa, M_w: 101 kDa, M_w/M_n (Đ): 2.0; batch

2: M_n: 40 kDa, M_w: 73 kDa, M_w/M_n (Đ): 1.8; batch 3: M_n: 45 kDa, M_w: 90 kDa, M_w/M_n (Đ): 2.0; batch 4: M_n: 113 kDa, M_w: 262 kDa, M_w/M_n (Đ): 2.3.

P3-F *- P(IF-dTdfBT)*

6,6,12,12-Tetraoctyl-6,12-dihydroindeno[1,2-b]fluoren-8-yl]-1,3,2-dioxaborolane diboronic ester (597.2 mg, 0.625 mmol), 4,7-bis(5-bromothiophen-2-yl)-5,6-difluoro-2,1,3-benzothiadiazole (**dTdFBT**) (309.0 mg, 0.625 mmol), $Pd(PPh_3)_4$ (14.5 mg, 0.013 mmol) and a stirrer bar were added to a 20 mL high pressure microwave reactor vial. The vial was then sealed with a septum and flushed with argon, before degassed toluene (3 mL), degassed aqueous 2M K_2CO_3 (1.1 mL) and two drops of aliquat-336 were added. The whole solution was then degassed again for 30 min before the reaction was heated to 120 °C for 3 days. To end-cap the polymer, the reaction was allowed to cool and a solution of phenyl boronic acid (6 mg in 0.2 mL toluene, 0.049 mmol) was injected and the reaction stirred for 2 h at 120°C. The reaction was again allowed to cool and bromobenzene (160 µL, 0.149 mmol) was then added and the reaction heated for a further 2 h. The mixture was then cooled to room temperature, precipitated in methanol (100 mL), stirred for 30 min and filtered through a Soxhlet thimble. The polymer was then extracted (Soxhlet) using methanol, acetone, hexane and $CHCl_3$ in that order under argon. The $CHCl_3$ fraction was collected and concentrated to ~70 mL, to which a solution of aqueous sodium diethyldithiocarbamate dihydrate solution (~100 mg in 70 mL) was added. The RBF was equipped with a condenser and the two layers were stirred vigorously at 60 °C for 60 min to extract the palladium. The two layers were then separated and the $CHCl_3$ layer washed thoroughly with water (3 x 100 mL). The $CHCl_3$ fraction was dried ($MgSO_4$), filtered and concentrated to ~10 mL before being precipitated into methanol (100 mL), stirred for 30 min and filtered. This precipitation was repeated again to yield P(IF-dTdFBT) as a pink/red solid (511 mg, 79%); M_n = 10.6 kDa, M_w = 21.7 kDa, M_w/M_n (Đ) = 2.05. ^{1}H NMR (400 MHz, $CDCl_3$) δ 8.40 – 8.28 (m, 2H), 7.85 – 7.53 (m, 10H), 2.20 – 1.97 (m, 8H), 1.67 – 1.47 (m, 8H), 1.20 – 1.00 (m, 40H), 0.79 – 0.71 (m, 12H); ^{19}F NMR (400 MHz, $CDCl_3$) δ -128.2.

P4-F *- P(BDTdTdFTz)*

Synthesis based on previously reported method[2]. 4,7-Bis(5-bromothiophen-2-yl)-2-(2-butyloctyl)-5,6-difluoro-2H-benzo[d][1,2,3]triazole (505.9 mg, 0.784 mmol), 2,6-bis(trimethyltin)-4,8-bis(5-(2-butyloctyl)thiophene-2-yl)-benzo[1,2-b;4,5-b']dithiophene (796.9 mg, 0.784 mmol), $Pd_2(dba)_3$ (14.3 mg, 0.016 mmol), P(*o*-tol)$_3$ (19.2, 0.063) and a stirrer bar were added to a 5 mL high pressure microwave vial. The vial was then sealed with a septum and flushed with argon, before degassed *o*-xylene (5 mL) was added. The solution was degassed for 10 min under argon. The vial was heated by microwave irradiation to 100 °C for 2 min, 140 °C for 2 min, 160 °C for 2 min, 180 °C for 10 min and

200 °C for 25 min. The solution was allowed to cool to room temperature, chlorobenzene (3 mL) was added to fully dissolve the polymer and the solution was precipitated into methanol (200 mL). The resulting suspension was stirred for 30 min and filtered through a Soxhlet thimble. The polymer was then extracted (Soxhlet) using methanol, acetone, hexane and chloroform in that order under argon. The chloroform fraction was concentrated to ~20 mL before being precipitated into methanol (200 mL), stirred for 30 min and filtered to yield **P4-F** as a black solid (760 mg, 86%); Mn of 37.9 kDa, Mw of 103.6 kDa, Mw/Mn (Đ) = 2.73; ^{1}H NMR (400 MHz, TCE-d2, 403K) δ 8.40 – 6.95 (m, 10H), 5.01 – 4.58 (m, 2H), 3.21 – 2.97 (m, 4H), 2.51 – 2.31 (m, 1H), 2.05 – 1.85 (m, 2H), 1.66 – 1.38 (m, 48H), 1.14 – 0.87 (m, 18H). ^{19}F NMR (400 MHz, $CDCl_3$) δ -133.0;

P5-F *– P(DTG-dTdVdfBT)*

(4,7-Bis[(E)-2-(5-bromo-3-dodecylthiophen-2-yl)ethenyl]-5,6-difluoro-2,1,3-benzothiadiazole) (137.9 mg, 0.156 mmol), 4,4-bis(2-octyldodecyl)-5,5-bis-(trimethyltin)-dithieno[3,2-b:2,3-d]germole (175.8 mg, 0.156 mmol), $Pd_2(dba)_3$ (2.6 mg, 0.0028 mmol), P(o-tol)$_3$ (3.8 mg, 0.011 mmol) and a stirrer bar were added to a 2 mL high pressure microwave reactor vial. The vial was sealed with a septum and flushed with argon, before degassed chlorobenzene (1 mL) was added. The whole solution was then degassed for 20 min under argon and the argon inlet removed. The vial was heated to 100 °C for 2 min, 140 °C for 2 min, 160 °C for 2 min, 180 °C for 10 min and 200 °C for 25 min. The polymer was cooled to RT and precipitated in methanol (100 mL), stirred for 30 min and filtered through a Soxhlet thimble. The polymer was extracted using Soxhlet apparatus (methanol, acetone, hexane) under argon to leave a dark blue polymer **P5-F** (220 mg, 93%); M_n: 27.9 kDa, M_w: 47.0 kDa, M_w/M_n (Đ): 1.69; ^{1}H NMR (400 MHz, $CDCl_3$, 50°C) δ 8.66 – 8.07 (br, 4H), 7.14 – 6.78 (br, 4H), 2.85 – 2.66 (br, 2H), 1.81 – 1.16 (m, 112H), 0.97 – 0.78 (m, 18H). ^{19}F NMR (400 MHz, $CDCl_3$) δ -134.8.

P6-F *– P(BDT-TT)*

Poly[4,8-bis(5-(2-ethylhexyl)thiophen-2-yl)benzo[1,2-b;4,5-b']dithiophene-2,6-diyl-alt-(4-(2-ethylhexyl)-3-fluorothieno[3,4-b]thiophene-)-2-carboxylate-2-6-diyl)] was purchased from Ossila; M_n: 21 kDa, M_w: 67 kDa, M_w/M_n (Đ): 3.3.

P1-SR-Suz

Synthesis and characterisation reported previously[1].

Synthesis of P1-SR, P2-Silane, P2-Copolymer, P3-SR, P4-SR, P5-SR and P6-SR

P1-SR

P1-F (3 mg, 0.004 mmol) and K_2CO_3 (100 mg, 0.72 mmol) were added to a 2 mL high pressure microwave vial. The vial was sealed with a septum and degassed with argon, before anhydrous chlorobenzene (0.75 mL) and DMF (0.25 mL) were added. Dodecanethiol (0.1 mL, 0.58 mmol) was then added and the solution was degassed with argon. The solution was heated in a microwave reactor at 120 °C for 30 min. After cooling the solution was precipitated into methanol (10 mL), stirred for 30 min and filtered through a Soxhlet thimble. Unreacted thiol was removed by washing (Soxhlet) with acetone and the polymer was extracted with $CHCl_3$. The $CHCl_3$ fraction was concentrated down to 1 mL and precipitated into methanol and filtered, resulting in a black solid (1.6 mg, 46%), M_n: 5.3 kDa, M_w: 6.2 kDa, M_w/M_n (Đ): 1.17; ^{1}H NMR (400 MHz, $CDCl_3$) δ 8.26 – 7.48 (m, 11H), 4.67 (br, 1H), 3.17 – 3.01 (m, 2H), 2.40 (br, 2H), 2.02 (br, 2H), 1.39 – 1.02 (m, 36H), 0.90 – 0.74 (m, 9H).

P2-Silane

P2-F (30 mg, 0.055 mmol), and K_2CO_3 (500 mg, 3.62 mmol) were added to a 20 mL high pressure microwave reactor vial. The vial was sealed with a septum and degassed with argon, before anhydrous chlorobenzene (4.5 mL) and DMF (1.5 mL) were added. (3-Mercaptopropyl)trimethoxysilane (0.1 ml, 0.54 mmol) was added and the solution degassed for 30 min. The solution was heated at 120 °C for 30 min in the microwave. After cooling the solution was precipitated into MeOH 100 mL and filtered. The solid was washed with MeOH and hexane to remove excess thiol and K_2CO_3. The yellow solid was then dried under vacuum to leave a yellow solid (30 mg, 76%); ^{1}H NMR (400 MHz, $CDCl_3$) δ 8.13 – 7.83 (m, 5H), 7.71 – 7.57 (m, 2H), 3.53 – 3.48 (m, 9H), 3.04 – 2.89 (m, 2H), 2.27 – 1.86 (m, 4H), 1.82 – 1.72 (m, 2H), 1.38 – 0.65 (m, 47H).

P2-Copolymer

P2-F (30 mg, 0.055 mmol), and K_2CO_3 (64 mg, 0.452 mmol) were added to a 5 mL high pressure microwave reactor vial. The vial was sealed with a septum and degassed with argon, before anhydrous chlorobenzene (2.25 mL) and DMF (0.75 mL) were added. Poly(2-ethyl-2-oxazoline) (α-benzyl, ω-thiol terminated, average M_n 2000 g/mol, Aldrich) (451.33 mg, 0.126 mmol) was then added and the solution was heated at 120 °C for 60 min in the microwave. After cooling the solution was precipitated into water 20 mL and filtered. Unreacted thiol polymer was removed by washing (Soxhlet) with acetone and the polymer was extracted into $CHCl_3$. The $CHCl_3$ fraction was concentrated down to 5 mL and precipitated into MeOH and filtered, resulting in a yellow solid (66 mg, yield 48%); ^{1}H NMR

(400 MHz, $CDCl_3$) δ 8.19 – 7.56 (m, 7H), 7.42 – 7.31 (m, 3H), 7.21 – 7.11 (m, 2H), 4.61 – 4.49 (m, 2H), 3.67 – 3.20 (m, 100H), 3.15 – 2.97 (br, 2H), 2.48 – 2.06 (m, 47H), 2.17 – 2.08 (br, 4H), 1.24 – 0.86 (br, 100H), 0.83 – 0.76 (br, 6H); IR 1632 cm^{-1} (C=O).

P3-SR

P3-F (9.5 mg, 0.009 mmol) and K_2CO_3 (100 mg, 0.72 mmol) were added to a 2 mL high pressure microwave vial. The vial was sealed with a septum and degassed with argon, before anhydrous chlorobenzene (0.75 mL) and DMF (0.25 mL) were added. Octanethiol (0.1 mL, 0.17 mmol) was then added and the solution was degassed with argon. The solution was heated in a microwave reactor at 120 °C for 30 min. After cooling the solution was precipitated into methanol (10 mL), stirred for 30 min and filtered through a Soxhlet thimble. Unreacted thiol was removed by washing (Soxhlet) with acetone and the polymer was extracted with $CHCl_3$. The $CHCl_3$ fraction was concentrated down to 1 mL and precipitated into methanol and filtered, resulting in a dark red solid (5.3 mg, 41%). M_n: 9 kDa, M_w: 14 kDa, M_w/M_n (Đ): 1.58; ^1H NMR (400 MHz, $CDCl_3$) δ 7.81 – 7.49 (m, 12H), 2.94 – 2.79 (m, 4H) 2.19 – 1.95 (m, 8H), 1.32 – 1.01 (m, 88H), 0.89 – 0.76 (m, 18H).

P4-SR

P4-F (10 mg, 0.009 mmol) was reacted using the same method for **P3-SR** to yield a black solid (6.7 mg, 51%). M_n: 37 kDa, M_w: 80 kDa, M_w/M_n (Đ): 2.17; ^1H NMR (400 MHz, TCE-d2, 403K) δ 8.13 – 7.99 (m, 2H), 7.86 – 7.75 (m, 2H), 7.50 – 7.35 (m, 4H), 7.05 – 6.96 (m, 2H), 4.80 – 4.60 (m, 2H), 3.05 – 2.92 (m, 8H), 1.91 – 1.24 (m, 90H), 1.04 – 0.89 (m, 25H).

P5-SR

P5-F (10 mg, 0.008 mmol), dodecanethiol (50 mg, 0.25 mmol) and K_2CO_3 (100 mg, 0.72 mmol) were added to a 5 mL high pressure microwave reactor vial. The vial was sealed with a septum and degassed with argon, before anhydrous chlorobenzene (3 mL) and DMF (0.75 mL) were added. The solution was stirred at 70 °C for 20 h. After cooling the solution was diluted with CH_2Cl_2 (20 mL) and washed with water to remove residual DMF and K_2CO_3. The solvent was then removed under reduced pressure. To remove the excess dodecanethiol the residue was repeatedly washed in refluxing acetone, resulting in a blue solid (6 mg, 47%); M_n: 28 kDa, M_w: 48 kDa, M_w/M_n (Đ): 1.69; ^1H NMR (400 MHz, $CDCl_3$ at 323K) δ 8.77 (d, *J* = 15.9 Hz, 2H), 7.91 (d, *J* = 15.9 Hz, 2H), 7.23 – 7.14 (m, 2H), 7.04 – 6.94 (m, 2H), 3.00 – 2.89 (m, 4H), 2.84 – 2.74 (m, 2H), 1.78 – 1.66 (m, 4H), 1.64 – 1.53 (m, 6H), 1.50 – 1.37 (m, 12H), 1.34 – 1.13 (m, 130H), 0.91 – 0.79 (m, 24H).

P6-SR

P6-F (9.7 mg, 0.011 mmol) was reacted using the same method for **P3-SR**, to yield a black solid (4.9 mg, 42%). M_n: 20 kDa, M_w: 66 kDa, M_w/M_n (Đ): 3.3 ; ^{1}H NMR (400 MHz, TCE-d2, 403K) δ 8.23 – 6.81 (m, 6H), 4.44 – 4.29 (br, 2H), 3.05 – 2.86 (m, 4H), 2.77 – 2.65 (br, 2H), 1.88 – 1.74 (m, 4H), 1.61 – 1.16 (m, 44H), 1.08 – 0.87 (m, 21H).

Substitution of P2-F with increasing thiol content

Reactant quantities and NMR characterisation for polymers with varying mol% of thiol:

20 mol%: **P2-F** (5.15 mg, 9.52 µmol), dodecanethiol (0.46 µL, 1.9 µmol); δ 3.02 – 2.93 (m, 0.4H).

40 mol%: **P2-F** (11.34 mg, 20.96 µmol), dodecanethiol (2.02 µL, 8.38 µmol); δ 3.02 – 2.93 (m, 0.8H).

60 mol%: **P2-F** (5.21 mg, 9.63 µmol), dodecanethiol (1.40 µL, 5.78 µmol); δ 3.02 – 2.93 (m, 1.2H).

80 mol%: **P2-F** (4.80 mg, 8.87 µmol), dodecanethiol (1.72 µL, 7.10 µmol); δ 3.02 – 2.93 (m, 1.6H).

100 mol%: **P2-F** (4.40 mg, 8.13 µmol), dodecanethiol (0.1 mL, 0.42 mmol); δ 3.02 – 2.93 (m, 2H). Anal. Calcd. for $C_{43}H_{58}N_2S_2$ C 77.42, H 8.46, N 4.20, found: C 77.33, H 8.64, N 4.33.

Synthesis of P2-N_3, P2-Alkene, P2-OR and P2-PEG

P2-N_3

P2-F (31 mg, 0.057 mmol, batch 1) and a stirrer bar was added to a 20 mL high pressure microwave vial. Chlorobenzene (4.5 mL) and DMF (1.5 mL) were added under ambient atmosphere and the solution was heated to 100 °C, when the polymer was fully dissolved a pellet of potassium hydroxide was then added. The solution was stirred for 10 min before adding *S*-(3-azidopropyl)thioacetate (20 µL, 0.14 mmol). The solution was heated for a further 10 min in the dark. The resulting solution was allowed to cool to room temperature before the addition of 5 mL $CHCl_3$. The organics were washed with water (2 x 30 mL), concentrated to 3 mL and precipitated into methanol (50 mL) and stirred for 30 min. Unreacted thiol was removed by washing (Soxhlet) with acetone and the polymer was extracted into $CHCl_3$. The $CHCl_3$ fraction was concentrated down to 1 mL and precipitated into methanol and filtered, resulting in a yellow solid (31 mg, 86%). M_n: 26.3 kDa, M_w: 42.4 kDa, M_w/M_n (Đ): 1.61. ^{1}H NMR (400 MHz, $CDCl_3$) δ 8.11 – 7.86 (m, 5H), 7.69 – 7.60 (m, 2H), 3.40 – 3.27 (m, 2H), 3.08 – 2.96 (m, 2H), 2.24 – 1.94 (m, 2H), 1.91 – 1.80 (m, 2H), 1.35 – 0.91 (m, 26H), 0.86 – 0.77 (m, 6H); IR 2094 cm^{-1} (-N=N=N).

P2-Alkene

P2-F (20.5 mg, 0.038 mmol, batch 3) was reacted using the same method as above, with *S*-(10-undecenyl) thioacetate instead of *S*-(3-azidopropyl)thioacetate to afford a yellow solid (13.5 mg, 46%). M_n: 51.2 k8Da, M_w: 86.3 kDa, M_w/M_n (Đ): 1.7. ^{1}H NMR (400 MHz, $CDCl_3$) δ 8.11 – 7.86 (m, 5H), 7.72 – 7.57 (m, 2H), 5.87 – 5.71 (m, 1H), 5.06 – 4.87 (m, 2H), 3.06 – 2.88 (m, 2H), 2.15 – 1.96 (m, 4H), 1.43 – 0.75 (m, 46H).

P2-OR

P2-F (23 mg, 0.043 mmol, batch 1), and a pellet of KOH were added to a 5 mL high pressure microwave reactor vial. The vial was sealed with a septum and degassed with argon, before anhydrous chlorobenzene (2.25 mL) and DMF (0.75 mL) were added. 2-ethyl-1-hexanol (0.067 mL, 0.430 mmol) was then added and the solution. The solution was heated at 130 °C for 60 min in the microwave. After cooling the solution was precipitated into methanol (20 mL) and filtered. Unreacted thiol was removed by washing (Soxhlet) with acetone and the polymer was extracted into $CHCl_3$. The $CHCl_3$ fraction was concentrated down to 4 mL and precipitated into methanol and filtered, resulting in a yellow solid (19 mg, 69%). M_n: 37 kDa, M_w: 56 kDa, M_w/M_n (Đ) 1.5. ^{1}H NMR (400 MHz, $CDCl_3$) δ 8.29 – 7.65 (m, 7H), 4.21 – 4.02 (br, 2H), 2.30 – 1.95 (br, 4H), 1.80 – 0.74 (m, 61H).

P2-PEG

P2-F (10 mg, 0.018 mmol, Batch 2), and KOH (1 pellet) were added to a 2 mL high pressure microwave reactor vial. The vial was sealed with a septum and degassed with argon, before anhydrous chlorobenzene (1.5 mL) and DMF (0.5 mL) were added. Triethylene glycol monomethyl ether (10uL, 1.6 mmol) then added and the solution. The solution was heated at 120 °C for 30 min in the microwave. After cooling the solution was precipitated into methanol (20 mL), filtered and washed with hot methanol. The polymer was then dried under vacuum overnight to leave a yellow solid (10.2 mg, 81%). M_n: 15.4 kDa, M_w: 28.4 kDa, M_w/M_n (Đ) 1.84. ^1H NMR (400 MHz, $CDCl_3$) δ 8.13 – 7.77 (m, 7H), 4.38 – 4.31 (br, 2H), 3.85 – 3.78 (br, 2H), 3.70 – 3.59 (m, 6H), 3.54 – 3.48 (br, 2H), 3.36 – 3.33 (br, 3H), 2.24 – 1.90 (br, 4H), 1.24 – 1.04 (m, 24H), 0.83 – 0.76 (br, 6H).

Supplementary Tables

Material	Linear Mobility (cm^2/Vs)	Saturation Mobility (cm^2/Vs)	Threshold Voltage (V)	$I_{ON/OFF}$
**P1-SR-Suz**	$1x10^{-4}$	$3x10^{-4}$	-38	10^{-4}
**P1-SR**	$0.8x10^{-4}$	$2.4x10^{-4}$	-52	10^{-4}

Supplementary Table 1: Properties of thin-film transistors of **P1-SR** and **P1-SR-Suz**

Equivalents	Reactive dye	Unreactive dye
423 (x)	2.4 µL, 2.4 nmol	2.4 µL, 2.4 nmol
827 (2x)	4.8 µL, 4.8 nmol	4.8 µL, 4.8 nmol
1714 (4x)	9.6 µL, 9.6 nmol	9.6 µL, 9.6 nmol
3429 (8x)	19.2 µL, 19.2 nmol	19.2 µL, 19.2 nmol
5143 (12x)	28.8 µL, 28.8 nmol	-
6857 (16x)	38.4 µL, 38.4 nmol	-

Supplementary Table 2: Equivalents of reactive and unreactive dye reacted with **SPN-N_3.**

Supplementary References

1. Creamer, A. *et al.* Systematic Tuning of 2 , 1 , 3-Benzothiadiazole Acceptor Strength by Monofunctionalization with Alkylamine , Thioalkyl , or Alkoxy Groups in Carbazole Donor − Acceptor Polymers. *Macromolecules* **50,** 2736–2746 (2017).

2. Casey, A. *et al.* Cyano substituted benzotriazole for use in organic solar cells. *J. Mater. Chem. A Mater. energy Sustain.* **5,** 6465–6470 (2017).

3. Deng, P. *et al.* Effect of bisalkylthio side chains on benzo[1,2-b:4,5-b′]dithiophene-based polymers for organic solar cells. *Dye. Pigment.* **138,** 47–55 (2017).

4. Fei, Z., Shahid, M., Yaacobi-gross, N. & Rossbauer, S. Thiophene fluorination to enhance photovoltaic performance in low band gap donor – acceptor polymers. *Chem. Commun.* 11130–11132 (2012). doi:10.1039/c2cc35079c

5. Casey, A. *et al.* Vinylene-Linked Oligothiophene−Difluorobenzothiadiazole Copolymer for Transistor Applications. *ACS Appl. Mater. Interfaces* **8,** 31154–31165 (2016).
